# Systematic review of the impact of restrictive wildlife trade measures on conservation of iconic species in southern Africa

**DOI:** 10.1111/cobi.14262

**Published:** 2024-04-05

**Authors:** Christina Hiller, Michael ’t Sas‐Rolfes

**Affiliations:** ^1^ Durrell Institute of Conservation and Ecology University of Kent Canterbury UK; ^2^ Oxford Martin Program on Wildlife Trade, University of Oxford, United Kingdom and African Wildlife Economy Institute Stellenbosch University Stellenbosch South Africa

**Keywords:** CITES, PRISMA methodology, prohibitions, SADC region, sustainable use, systematic review, wildlife economy, wildlife trade, CITES, comercio de vida silvestre, economía de vida silvestre, metodología PRISMA, prohibiciones, región SADC, revisión sistemática, uso sustentable

## Abstract

Trade restrictions are often advocated and implemented as measures to protect wild species threatened by overexploitation. However, in some instances, their efficacy has been questioned, notably by governments in the southern African (SADC) region, which tend to favor a sustainable use approach to wildlife management. We conducted a systematic review of published literature guided by the PRISMA process to examine the effectiveness of trade restrictions and directly related control measures in addressing threats to species conservation in the SADC region, with a focus on elephants (*Loxodonta* sp.), rhinoceroses (*Ceratotherium simum*, *Diceros bicornis*), lions (*Panthera leo*), and pangolins (*Manis* sp.). We focused in particular on the direct conservation impact of trade restrictions at species or population level, indirect conservation impact at human behavior or attitude level, and socioeconomic impact on rural livelihoods and well‐being and on national economies. Research on these topics was uneven and focused strongly on the effects of trade restrictions and law enforcement on crime‐related behavior. Research gaps include socioeconomic impacts of trade restrictions, including effects of international restrictions on local livelihoods and consequent secondary conservation impacts, and evaluations of attempts to disrupt criminal networks. Based on the reviewed impact evidence, the effectiveness of international trade restrictions depends on a range of fully aligned measures in countries of origin, transit, and consumption. For example, our results suggest positive ecological short‐term but negative or unknown long‐term socioeconomic impacts of domestic restrictions. Based on these findings, key policy requirements include more nuanced approaches to incorporate a range of appropriate measures in range, transit, and consumer countries, that focus on capacity development for early detection and apprehension of incursions inside protected areas; measures for constructive engagement with relevant local communities outside protected areas; and future research to improve understanding of the socioeconomic contribution of wildlife.

## Abstract (es)

Revisión sistemática del impacto de las medidas restrictivas de comercio de vida silvestres sobre la conservación de especies icónicas en el sur de África

**Resumen:**

Las restricciones comerciales a menudo se promueven e implementan como medidas para proteger a las especies silvestres amenazadas por la sobreexplotación. Sin embargo, en algunas instancias, su eficacia ha sido cuestionada, en particular por gobiernos de la región del sur de África (SADC), que tienden a favorecer un enfoque de uso sustentable para el manejo de la vida silvestre. Realizamos una revisión sistemática de la literatura publicada guiada por el proceso PRISMA para examinar la efectividad de las restricciones comerciales y las medidas de control directamente relacionadas para enfrentar las amenazas a la conservación de especies en la región de la SADC, con un enfoque en elefantes (*Loxodonta sp*.), rinocerontes (*Ceratotherium simum*, *Diceros bicornis*), leones (*Panthera leo*) y pangolines (*Manis* sp.). Nos centramos en particular en el impacto directo sobre la conservación de las restricciones comerciales a nivel de especie o población, el impacto indirecto sobre la conservación a nivel de comportamiento o actitud humana, y el impacto socioeconómico en los medios de vida y el bienestar rurales y en las economías nacionales. La investigación sobre estos temas fue desigual y se centró principalmente en los efectos de las restricciones comerciales y la aplicación de la ley en el comportamiento relacionado con el delito. Los vacíos a investigar incluyen los efectos de las restricciones internacionales sobre los medios de vida locales y los consiguientes impactos secundarios en la conservación, y evaluación de los intentos de desarticular las redes criminales. Con base en la revisión de la evidencia del impacto, la efectividad de las restricciones de comercio internacional depende de una gama de medidas totalmente alineadas en los países de origen, tránsito y consumo. Por ejemplo, nuestros resultados sugieren impactos socioeconómicos ecológicos positivos a corto plazo, pero negativos o desconocidos a largo plazo, de las restricciones nacionales. Con base en estos hallazgos, los requisitos clave para la definición de políticas incluyen enfoques más matizados para incorporar una variedad de medidas apropiadas – en los países de origen, tránsito y consumidores – que se centren en el desarrollo de capacidades para la detección temprana y la detención de incursiones dentro de áreas protegidas; medidas para un compromiso constructivo con las comunidades locales relevantes fuera de las áreas protegidas; e investigaciones futuras para mejorar la comprensión de la contribución socioeconómica de la vida silvestre.

## INTRODUCTION

Wild populations of iconic species, such as elephants (*Loxodonta* sp.), rhinoceroses (*Ceratotherium simum*, *Diceros bicornis*), lions (*Panthera leo*), and pangolins (*Manis* sp.), have been receding across many parts of Africa and are reported to be threatened by poaching for trade purposes (Bauer et al., 2016; Emslie, 2020; Emslie et al., 2016; Gobush et al., 2022; Nixon et al., 2019; Pietersen et al., 2019). Accordingly, various policy measures seek to address the perceived threat from trade. However, due to varying success rates, such measures are regularly debated. It is, thus, imperative to evaluate this threat and current attempts to mitigate it in relation to other factors that may drive wildlife decline. More specifically, there is a need to consider the effectiveness of wildlife trade restrictions as a conservation measure in the southern African (SADC) region.

Historical events have shaped the perception of trade activities as main threats to southern African wildlife, including the adoption of trade restrictions to address these. Excessive hunting for meat, other products, and sport in North America and southern Africa, especially during the second half of the 19th century, led to growing awareness of overhunting as a threat to species survival and various consequent attempts to prevent this (Beinart & Coates, 2002). However, the perception of trade as a threat to species developed differently in the 2 regions, eventually resulting in significantly divergent wildlife governance models.

The North American public associated the destruction of species, such as bison (*Bison bison*) and passenger pigeon (*Ectopistes migratorius*), with market hunting and trade, which resulted in trade‐restrictive legislation in the form of the Lacey Act as early as 1900. In contrast, after a period of consolidation through area‐based state protection, southern Africa began to embrace market‐based approaches to wildlife governance with the onset of game ranching in the 1960s. Southern African countries thus became policy outliers, allowing and even encouraging sustainable use and trade in wildlife predicated on the devolution of wildlife proprietorship and use rights (including the right to trade), which underpin the region's contemporary sustainable use approach (Abensperg‐Traun, 2009). In most other countries, wildlife is state‐owned or an open‐access resource. The corresponding absence of rights and capacities of exclusion conferred by devolved proprietorship calls for mechanisms, such as trade restrictions, to prevent overexploitation (Schlager & Ostrom, 1992).

The early 20th‐century US policy approach to wildlife trade gradually extended internationally and, embodied by a 1963 resolution by the International Union for Conservation of Nature (IUCN), provided the impetus for the Convention on International Trade in Endangered Species of Fauna and Flora (CITES), effective from 1975. The agreement aims to regulate trade across international boundaries by establishing protocols between signatory countries. It employs a listing mechanism whereby all international trade in species listed on CITES Appendix II is subject to a permitting requirement to deter unsustainable levels of exploitation. Species considered threatened with extinction are listed in Appendix I, and international commercial trade in these species is prohibited. Notwithstanding these listings, countries can apply stricter domestic measures (e.g., prohibition of imports or exports of Appendix II species). Crocodilians and spotted cats were initial concerns for CITES in the late 1970s (Wijnstekers, 2018).

**FIGURE 1:**
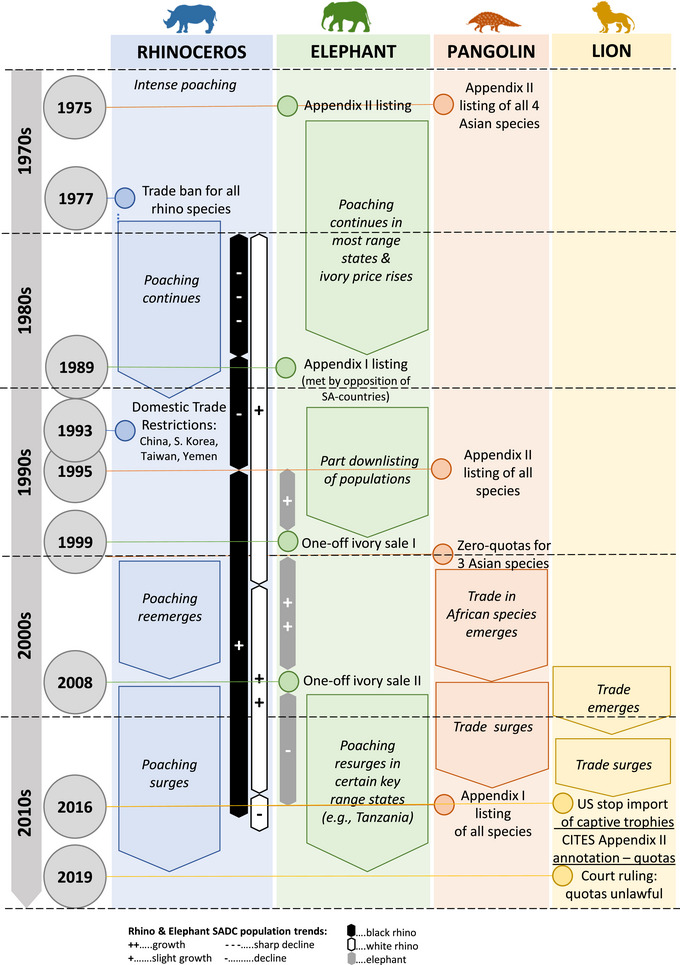
Trade‐restrictive policy interventions and related indicative trends for 4 selected iconic species in the southern African (SA) region over the last 50 years before the COVID‐19 pandemic (1970–2020).

Given our focus on elephants, rhinoceroses, pangolins, and lions, Figure 1 provides an overview of CITES‐related trade‐restrictive policy interventions and related indicative trends for these species from 1970 to 2020. Rhinoceroses and elephants drew attention as overexploited species for trade purposes early on (Martin, 1983; Somerville, 2016). More recently, pangolin species have similarly gained prominence (Heinrich et al., 2016), and there are growing concerns about possible trade impacts on African lions (*Panthera leo*) (Williams et al., 2017a). A surge in poaching and trafficking of these species in the late 2000s (Figure 1) (and the concomitant emergence of a new legal export trade of lion body parts from South Africa) prompted governments and nongovernmental organizations (NGOs) to take action, which included promoting and implementing further trade restrictions (’t Sas‐Rolfes, 2017). These responses are ongoing, and wildlife trade (both illegal and legal), especially in the charismatic mammalian species, features as a controversial and fiercely debated issue on global conservation policy and research agendas. Although CITES provides an overall framework for regulating or restricting trade, governments have employed a range of additional restrictive or restriction‐supportive policy measures throughout the trade chain, including law enforcement and efforts to reduce consumer demand through behavior change interventions.

Although shrinking, degrading, and fragmenting habitat; human intrusion; loss of prey (for carnivores); and associated human–wildlife conflict, all possibly aggravated by climate change, remain key underlying drivers of wildlife loss, wild populations of rhinoceroses, elephants, pangolins, and various other species are at least in some instances also threatened by excessive targeted harvesting for trade purposes, according to IUCN Red List assessments (which draw on expert opinion to appraise pressures on species). To date, debates persist over which policy interventions address the full suite of threats to southern Africa's wildlife most effectively, holistically, and synergistically. The effectiveness of trade restrictions features prominently in such debates. Given that internationally imposed restrictions potentially constrain the sustainable use model of wildlife management favored by southern African governments, they must be evaluated carefully.

With this in mind, we conducted a comprehensive and systematic review of scientific and gray literature to provide a clear and structured synthesis of the existing evidence on the impact and effectiveness of international wildlife trade restrictions for conservation in SADC countries from 1970 to 2020. The review purposefully excluded any developments during the COVID‐19 pandemic due to its unique and substantial influence on trade patterns, incomparable to the previous decades. Our results are based on a project funded by the U.S. Agency for International Development (USAID) with a broader focus (but limited to the specified iconic species), including measures aimed at indirectly influencing or controlling trade, such as resource rights regimes (’t Sas‐Rolfes & Hiller, 2020). The review framework builds on earlier work that evaluated the conservation effectiveness of trade‐related policy interventions at the global level (Cheng et al., 2017; UNEP, 2019) but concentrates more specifically on the impact of trade restrictions on the conservation of elephants, rhinoceroses, pangolins, and lions in SADC countries.

## METHODS

We analyzed and synthesized the results of 46 studies that qualified as impact assessments of trade restrictions undertaken from 1970 to 2020 guided by the Preferred Reporting Items for Systematic Reviews and Meta‐Analyses framework (PRISMA) (Moher et al., 2009). We selected the studies based on an analytical framework for evaluating wildlife trade restrictions on conservation outcomes and employed a rigorous review protocol, as described below and guided by previous relevant literature (Galvin et al., 2018; Partelow et al., 2017; Soliku & Schraml, 2018).

### Analytical framework

**FIGURE 2:**
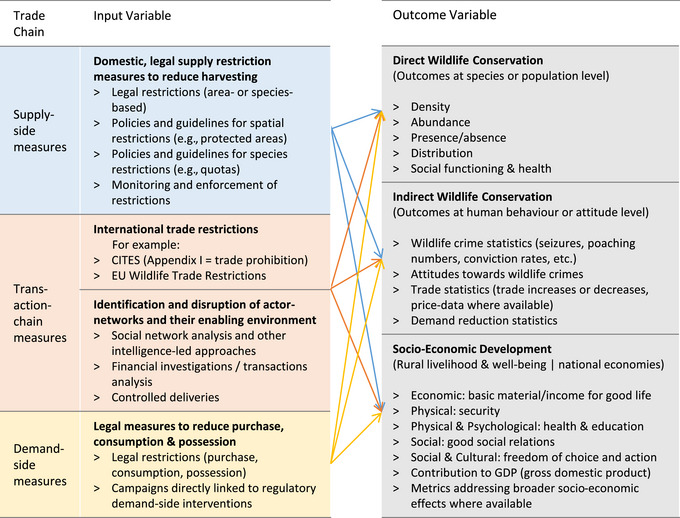
Analytical framework for assessing the existing evidence regarding the impact and effectiveness of wildlife trade restrictions at reducing threats to iconic wildlife species in terms of wildlife conservation and socioeconomic outcomes in SADC countries (Angola, Botswana, Namibia, Malawi, Mozambique, South Africa, Tanzania, Zambia, and Zimbabwe).

We initially developed a basic analytical framework of influencing and outcome variables consistent with the review scope (Figure 2). The framework shows the influencing factors on the left of the diagram and the outcome variables on the right. The input factors were structured according to a simplified trade chain (’t Sas‐Rolfes et al., 2019). We synthesized categories used in previous frameworks to account for relevant impact factors along these trade‐chain dimensions (Cheng et al., 2017; UNEP, 2019).

The input variables constituted restrictive legal measures targeted at activities along the trade chain to curb or prohibit trade in wildlife species. Further institutional and governance factors, while influencing the ability to trade, were excluded from the framework. Such factors included property and resource rights regimes and other supporting measures (i.e., provision of legal supply substitutes, product traceability measures, and voluntary behavior change initiatives).

We clustered the outcome variables on the right‐hand side of the analytical framework into findings about the direct and indirect impacts and effectiveness of wildlife trade restrictions for conserving wildlife. Direct impacts included outcomes at the species or population level. Indirect impacts encompassed outcomes on a human behavior or attitude level because they were used to infer wildlife crime or demand‐related effects. Even further removed, but still potentially relevant as indirect influences on conservation outcomes (and therefore included in the analysis), were socioeconomic development factors, such as contributions to local livelihoods and well‐being and those to national economies.

### Literature search and selection

We systematically searched peer‐reviewed and gray wildlife trade literature published from 1970 to 2020 in English across the following countries: Angola, Botswana, Namibia, Malawi, Mozambique, South Africa, Tanzania, Zambia, and Zimbabwe. Starting the search in 1970 ensured coverage of all publications assessing the impact of international trade restrictions linked to the ratification of CITES in 1975 and the years leading up to its inception. We chose 2020 as the cutoff date for the search to exclude results that might be influenced by the effects of COVID‐19 on global and local trade.

Guided by the analytical framework, we conducted an extensive search on Web of Science (6 May 2020), Scopus (8 May 2020), and Google Scholar (19 May 2020). We used the Boolean search terms (Table 1), specifying geographical areas, animal species, and trade‐restriction‐related keywords. Our search further targeted specialist websites and databases during the specified time frame to account for relevant nonpeer‐reviewed studies (i.e., gray literature, including reports of NGOs, government agencies, or other relevant institutions) (Appendix S1). Where appropriate, we applied a snowball approach during our screening process (i.e., using references of selected papers to find additional sources) and combined all search results into a single data set. Compared with peer‐reviewed literature, it was impossible to conduct gray literature searches with the same rigor. Hence, our results might not represent all evidence‐based data available at the point of the literature search.

**TABLE 1:** Search strings and procedures for the search of the peer‐reviewed literature on measures targeted to restrict wildlife trade to reduce threats to iconic wildlife species, specifically elephants, rhinoceroses, lions, and pangolins in terms of wildlife conservation and socioeconomic outcomes in Angola, Botswana, Namibia, Malawi, Mozambique, South Africa, Tanzania, Zambia, and Zimbabwe (SADC countries).

Web of Science Boolean search string
TS = (“SADC” OR “Southern African Development Community” OR “south* Africa*” OR “Angola” OR “Botswana” OR “Namibia” OR “Malawi” OR “Mozambique” OR “South Africa” OR “Tanzania” OR “Zambia” OR “Zimbabwe”) AND TS = (“African elephant” OR “Loxodonta africana” OR “rhino*” OR “white rhino*” OR “Ceratotherium simum” OR “black rhino*” OR “Diceros bicornis” OR “lion” OR “Panthera leo” OR “pangolin” OR “Smutsia temminckii” OR “Smutsia gigantea” OR “wildlife” OR “conservation” OR “fauna” OR “ivory”) NOT TS = (“flora” OR “fish*” OR “tree*” OR “*aqua*” OR “timber”) AND TS = (“wildlife” NEAR/3 “crime” OR “demand” OR “product*” OR “consum*” OR “trade” OR illegal NEAR/5 “wildlife trade” OR illicit NEAR/5 “wildlife trade” OR “traffic*” OR “poach*”) NOT TS = trade‐off AND TS = (“law” OR “legal” OR “legislat*” OR “restrict*” OR “policy” OR “regulat*” OR “*guideline*” OR “*complian*” OR “*quota*” OR “*enforce*” OR “*monitor*” OR “*prosecut*” OR “CITES” OR “Convention on International Trade in Endangered Species” OR “moratorium” OR “*ban*” OR “*ban*” NEAR/3 (“demand reduc*” OR “campaign*”) OR “land owner*” OR “protected area*” OR “resource right*” OR “community” NEAR/5 (“wildlife” OR “resource” OR “steward*”) OR “state‐owned” NEAR/3 (“land” OR “resource*”) OR “actor” NEAR/3 “network” OR “social network analysis” OR “investigat*”)
SCOPUS Boolean search string
Search 1: TITLE‐ABS‐KEY(SADC OR “Southern African Development Community” OR “south* Africa*” OR Angola OR Botswana OR Namibia OR Malawi OR Mozambique OR South Africa OR Tanzania OR Zambia OR Zimbabwe) Search 2: TITLE‐ABS‐KEY(“African elephant” OR “Loxodonta africana” OR rhino* OR “white rhino*” OR “Ceratotherium simum” OR “black rhino*” OR “Diceros bicornis” OR lion OR “Panthera leo” OR pangolin OR “Smutsia temminckii” OR “Smutsia gigantea” OR wildlife OR conservation OR fauna OR ivory) AND NOT TITLE‐ABS‐KEY(flora OR fish* OR tree* OR *aqua* OR timber) Search 3: TITLE‐ABS‐KEY(wildlife OR crime OR demand OR product* OR consum* OR “wildelife trade” OR illegal OR illicit OR traffic* OR poach*) AND NOT TITLE‐ABS‐KEY(trade‐off) Search 4: TITLE‐ABS‐KEY(law OR legal OR legislat* OR restrict* OR policy OR regulat* OR *guideline* OR *complian* OR *quota* OR *enforce* OR *monitor* OR *prosecut* OR CITES OR {Convention on International Trade in Endangered Species} OR moratorium OR *ban* OR “land owner*” OR “protected area” OR “resource right” OR community OR state?owned OR resource* OR network OR {social network analysis} OR investigat*) Combine search sets in search history: search 1 AND search 2 AND search 3 AND search 4
Google Scholar Boolean search string (Procedure: Title screening of first 900 papers and extension of data set with new studies that could not be rejected based on their title.)
illegal wildlife trade law OR legal OR legislat* OR restrict* OR policy OR *quota* ‐flora ‐fish ‐tree ‐aqua ‐timber

*Note*: The search strings are the originals used for the project funded by USAID with a broader focus, including measures aimed at indirectly influencing or controlling trade, such as property and resource rights regimes. For this review, we included only the search results within the boundaries of the framework in the “Analytic framework” section (i.e., the impact of trade restrictions in SADC countries, with a further focus on conserving elephants, rhinoceroses, pangolins, and lions).

**FIGURE 3:**
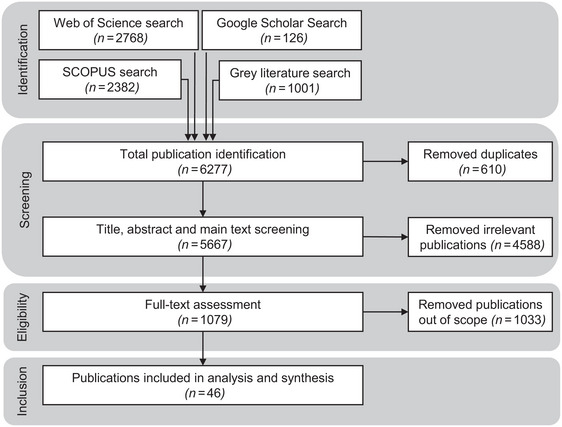
Information flow through the systematic step‐wise approach to screen and review articles resulting from searches of the literature on evidence‐based studies illustrating the impact and effectiveness of governance measures that restrict wildlife trade for reducing threats to iconic wildlife species, specifically elephants, rhinoceroses, lions, and pangolins, and socioeconomic development in SADC countries (Angola, Botswana, Namibia, Malawi, Mozambique, South Africa, Tanzania, Zambia, and Zimbabwe).

After implementing the search strategy and eliminating duplicates, all papers (originally 5667 without duplicates) were subjected to a multistep screening process, initially screening titles, then abstracts, and finally evaluating full publications (Figure 3). We excluded all articles unrelated to our study topic, outside the geographical location, ineligible publication types (e.g., editorials, commentaries, etc.), and papers not in English during the title‐screening phase. During abstract screening, studies incompatible with the analytical framework were removed. Finally, we read the main texts and rejected all papers that did not qualify as evidence‐based impact evaluations. We only included experimental and quasi‐experimental studies that analyzed cause‐and‐effect relationships and considered confounding factors, along with qualitative studies that applied rigorous validity and reliability measures such as triangulation or intercoder reliability, in addition to systematic reviews and multiregression models testing the sensitivity of results to potential confounders. In other words, we excluded publications that did not examine the effects of at least one influencing factor on at least one outcome variable of the framework beyond monitoring approaches (Cheng et al., 2017) or exhibited no counterfactual thinking (Baylis et al., 2016). This screening process resulted in a total of 46 accepted studies.

### Data analyses

For data analyses and synthesis purposes based on qualitative thematic analysis (Braun & Clarke, 2006), we prepared a coding sheet and data extraction protocol (Appendix S2) and added relevant information to the data set in Microsoft Excel 2110 accordingly. We ascertained evidence‐based research for each combination of influencing factors and outcome variables, resulting in clusters of studies for these combinations with reference to species and countries. We used open, manual coding to analyze this qualitative data inductively, inferring evidence for the impacts of influencing factors on outcome variables (Drury et al., 2011; Newing et al., 2011). We further synthesized the themes of findings and insights of the study clusters by grouping similar codes together to form themes (Gioia et al., 2013; Khan, 2014). The themes described particular levers or determinants wielding a specific effect on outcome variables. Based on these levers, we refined our synthesis through verbal discussions relating and contrasting the themed insights from the various studies, resulting in an organized overview of the existing research (Tables 2, 3, 4).

**TABLE 2:** Themes of supply‐side and transactional measures to restrict trade in iconic species, specifically elephants, rhinoceroses, lions, and pangolins, with corresponding research findings from evidence‐based impact studies illustrating the impact and effectiveness of governance measures to restrict wildlife trade at reducing threats to iconic wildlife species in terms of direct conservation outcomes on a species or population level and indirect conservation outcomes on a human behavior level with a focus on Angola, Botswana, Namibia, Malawi, Mozambique, South Africa, Tanzania, Zambia, and Zimbabwe.

	Input	Output: Impact on Code them Wildlife conservation: direct (species & populations) & indirect (human behavior)	
Supply‐side measure	Domestic laws, policies, and regulations to reduce harvesting	S‐C1	Hunting restrictions merely short‐term measures
			Lion: 3‐year hunting moratorium in Zambia (2013–2015): Increase of lion population in Luangwa National Park; markedly higher adult male survival rate Risk of uncontrolled meat poaching and human encroachment by rural communities due to loss in livelihood and human well‐being in cases of longer‐term restrictions (Mweetwa et al., 2018; Rosenblatt et al., 2014). Elephant: Prohibition of elephant hunting in Botswana (introduced 2014): Poaching intensified significantly between 2014 and 2018 (Schlossberg et al., 2019).General: Hunting moratorium of 2014 in Botswana: Progressively antagonistic attitudes of towards wildlife of villagers of community‐based natural resource management programs (CBNRM) and perceived rise of poaching levels (Gaodirelwe, Masunga, et al., 2020; Gaodirelwe, Motsholapheko, et al., 2020).
		Direct wildlife conservation: species & populations	
		S‐C2	Uncoordinated cross‐border hunting restrictions disturb animal social structures
			Elephant: Unaligned, individual hunting quotas in Greater Mapungubwe Transfrontier: Conservation Area between Botswana, South Africa, and Zimbabwe: Loss of trophy bulls with expected detrimental effects on social structures (Selier et al., 2013).
		Indirect wildlife conservation: human behavior	
		S‐C3	More behavior change through permission instead of prohibition regulations
			Lion: Age‐based “bonus‐malus” system for lion trophy hunter behavior in Mozambique: Incentivizes hunters toward older trophies; reduced risk of hunting young, reproducing male lions; fewer trophies per annum overall (Begg et al., 2018).
Supply‐side measures	Monitoring and enforcement	S‐C4	Sufficient high‐quality and proactive enforcement serves wildlife conservation
			DIRECT CONSERVATION IMPACT (Species & Populations) Elephant: Markedly increased law enforcement efforts and investigation operations: Stabilized elephant numbers in Luangwa Valley in Zambia (Jachmann & Billiouw, 1997). Lion: Reserves with the largest management budget: Highest lion populations (Packer et al., 2013). Rhinoceros & Elephant: Small antipoaching field staff units in Zambia's Luangwa Valley (low manpower) and mild prison sentences for offenders: Unabated rhinoceros and elephant population declines due to illegal activity (1979–1985); effective at capturing poachers, but ineffective at protecting the large elephant populations over the vast area (Leader‐Williams, 1996; Leader‐Williams et al., 1990).
			INDIRECT CONSERVATION IMPACT (Human Behavior) Elephant: Government effectiveness and human development: Impact on poaching levels measured as the Proportion of Illegally Killed Elephants (PIKE) at the country level (Burn et al., 2011). Elephant: Significant increases of law enforcement staff in Zimbabwe's Gonarezhou National Park between 2000 and 2010: Minimized illegal hunting, despite an expected increase in poaching due to the country's economic collapse (Gandiwa et al., 2013). Rhinoceros: Law enforcement efforts in the Lower Zambezi Valley in Zimbabwe: No apparent reduction in illegal activity with estimates that a 2% elephant population growth rate needs triple the field staff (Martin, 1993). Rhinoceros: Rapid response measures: identified as a suitable means to recover rhinoceros horns (Milledge, 2007). Rhinoceros: Generalized linear model factors (i) GDP per capita in Far East Asia, (ii) fine upon conviction, and (iii) antipoaching efforts most sensitive predictors of: Rhinoceros poaching levels in South Africa, emphasizing suitable law enforcement measures as vital to curb rhinoceros poaching under increasing wealth scenarios in the Far East (Di Minin et al., 2015). Rhinoceros: Focus on leadership and intelligence, with sufficient funding most influential for: low rhinoceros poaching levels in a Zimbabwean reserve, largely due to highly committed, loyal, and long‐term staff workforce limiting the access of poachers to inside knowledge of security systems and flipping antipoaching activities from reactive to proactive (Ball et al., 2018). General: Increased capacity and capability of well‐paid law enforcement village scouts with superior knowledge of the surrounding area in Zambia's Lupande Game Management Area: Dramatic increases in arrests and firearm confiscations; more protective attitude of villagers for wildlife (Lewis et al., 1990). General: First 10 days of patrol periods of patrol teams: Most effective for encountering illegal activities and making arrests (Siamundaala et al., 2009).
Supply‐side measures	Monitoring and enforcement	S‐C5	effective law enforcement hinges more on detection risk than harm or loss
			Elephant/Rhinoceros: Strong law enforcement more effective than variable or fixed penalties according to an economic model on black rhinoceroses and elephants in Zambia's Luangwa Valley (1979–1985) to deter organized crime gangs because potential penalties for poaching would need to be raised exponentially to counter the vast gains, including a shoot‐to‐kill policy to constitute an effective deterrent (Leader‐Williams & Milner‐Gulland, 1993; Milner‐Gulland & Leader‐Williams, 1992). Elephant: The probability of interception strikingly more effective than lowering black market ivory prices according to a bioeconomic model to: reduce the planned number of poaching expeditions (Lopes, 2015). Pangolin: Stark increase in the number of confiscated ground pangolins (*Smutsia temminckii*) in Zimbabwe (2010–2015) combined with severe penalties: pangolins taken from the wild for the black market limited (Shepherd et al., 2017). General: Dramatic increase of poaching costs (willingness to implement shoot poachers on sight policies) according to an economic model of poaching constitutes: The only way to protect endangered species when nonpoaching wages are low and the economic benefits of poaching are high (Messer, 2010). General: Law enforcement/park ranger presence, dangerous wildlife and fines in Tanzania: Negligible probabilities of any harm (fines or injury) when hunting illegally; perceived higher number of hours a poacher needs to remain concealed to avoid detection (Knapp, 2012).
		S‐C6	Early‐stage new technologies to enhance detection rates
			Elephant: Based on DNA‐matching technology poaching affects: the social structure of elephant populations (Mondol et al., 2014). General: New technologies such as thermal imaging (Hart et al., 2015), remotely piloted aircraft systems fitted with cameras, AI‐based applications (Bondi et al., 2018; Mulero‐Pázmány et al., 2014), and GIS and remote sensing to monitor poaching activities (Sibanda et al., 2016) suggest: Advanced poaching detection rates under experimental conditions.
		Indirect wildlife conservation: human behavior	
		S‐C7	Need to couple enforcement with further measures
			Elephant: Continued investment in law enforcement needs to be combined with interventions targeting ivory demand reduction, corruption, and poverty alleviation to: reduce elephant poaching (Hauenstein et al., 2019). Elephant: Levels of regional legal protection and other factors, in particular human access, the corruption perception index, and ecosystem type being affected: elephant poaching levels (CITES Secretariat, 2007). Rhinoceros: Insufficient or complicit law enforcement in South Africa attributed to explain: higher levels of patrol efforts around poaching hotspots before rhinoceros poaching incidents, that is, field rangers were at the right place, but at the wrong time, suggesting that even high presence in a poaching area does not deter organized rhinoceros poaching gangs (Barichievy et al., 2017). General: Regular monitoring of animal populations to: identify poaching hotspots and worrying trends as a crucial element for law enforcement success (Bauer et al., 2015; Ferreira et al., 2017).
		S‐C8	Influence of nonmarket related social constructs on law enforcement effectiveness
			Rhinoceros: The relationship constructs of societies with the rhinoceros beyond a rational, economical approach result in: different levels of moral protection of the species, thereby influencing the effectiveness of antipoaching measures (Tanghe, 2017).
Transactional measures	International trade restrictions: CITES Appendix I listings	T‐C1	Trade bans' tendency for law enforcement evasion and driving markets underground
			Rhinoceros: CITES Appendix 1 Listing: Horns increasingly evading law and enforcement reaching illegal markets (from 2001) (Cheteni, 2014). Rhinoceros: CITES Appendix 1 Listing followed by additional legal restrictions in South Africa: escalation of illegally killed rhinoceroses from 2007 despite substantial supporting law enforcement efforts (Emslie et al., 2016; Ferreira et al., 2018). Pangolin: Immediate onset of CITES imposed zero‐quota policy in 2000: a spike in pangolin trade in Asia; a clear transfer of the preban trade volume to illicit markets (Challender et al., 2015). Lion: Anticipated restrictive (zero) export quota for lion products, imposed at CITES CoP17: surge in legal exports of lion skeletons from South Africa in 2016 and rising concerns of emerging black markets for lion products (Williams et al., 2017a). Elephant: restricted ivory supply due to CITES Appendix I listing (1989) compounded by Chinese ivory trafficking interception measures (2011–2012): quick rise of illegal ivory prices accelerating elephant poaching rates (Zhou et al., 2018).
		T‐C2	Unregulated markets, widespread corruption, civil unrest or poor wildlife management increase likelihood of trade ban ineffectiveness
			Elephant: CITES Appendix‐I listing (1989): Short‐term reduction of poaching pressure on many populations (Stiles, 2004; Zhou et al., 2018); however, persistent poaching increasing markedly from 1998 in countries with widespread corruption, civil unrest, or poor wildlife management, leading to further declining elephant numbers, particularly in West and Central Africa (Stiles 2004; Underwood et al., 2013). Elephant: CITES ivory ban: reversing overall decline of African elephant numbers between 1989 and 2007, except in countries with unregulated postban ivory markets (Lemieux & Clark, 2009).
		T‐C3	Potential for trade bans' short‐term effectiveness
			Elephant: CITES Appendix‐I listing (1989): short‐term reduction of poaching pressure on many populations (Lemieux & Clark, 2009; Stiles, 2004; Underwood et al., 2013; Zhou et al., 2018). Modeling approaches suggested that the effectiveness of trade restrictions hinges on further conditions (Heltberg, 2001; Khanna & Harford, 1996).
	Actor‐network disruption	Wildlife conservation: indirect (human behavior)	
		T‐C4	Lack of collaborative approaches across jurisdictions
			Rhinoceros: Social network analyses for trafficking network close to Kruger National Park: Crucial element to disrupt transnational trafficking networks effectively (Haas & Ferreira, 2015). Rhinoceros: Lack of coordinated intelligence activities and inadequate legislation for prosecution: organized crime dealers and kingpins favor efficiency over security, as the risks of being caught and prosecuted are minimal, and security plans for crime syndicate members and their families exist (Hübschle, 2017).

*Note*: Theme codes (e.g., S‐C3) are referenced in “Results” in parentheses.

**TABLE 3:** Themes of supply‐side and transactional measures to restrict trade in iconic species, specifically elephants, rhinoceroses, lions, and pangolins, with corresponding research findings from evidence‐based impact studies illustrating the impact and effectiveness of governance measures that restrict wildlife trade at socioeconomic development in terms of rural livelihoods and well‐being and national economies with a focus on Angola, Botswana, Namibia, Malawi, Mozambique, South Africa, Tanzania, Zambia, and Zimbabwe.

	Input	Output: Impact on Code theme Socioeconomic development: rural livelihoods & well‐being	
Supply‐side	Domestic legislation	S‐SE1	Hunting restrictions harmful to rural livelihoods
			General: Antihunting regulations in Namibia and Mozambique: negative attitudes of most study participants due to wide‐ranging negative effects on their well‐being (food, assets, direct income [from illegal rhinoceros hunting]) (Strong & Silva, 2020). General: hunting moratorium of 2014 in Botswana: negative effects on household well‐being due to increased crop‐raiding and predation, job losses, and decline in income (Gaodirelwe, Masunga, et al., 2020; Mbaiwa, 2018). General: Zambian hunting moratorium (2013–2014): adverse livelihood and well‐being effects in already poverty‐stricken areas due to lost meat and (seasonal) income possibilities through sport hunting (White & Belant, 2015). General: Restrictive hunting legislation in Botswana: unwillingness of the Basarwa to submit to external controls over wildlife use and hunting, as wildlife is part of Basarwa's diet due its association with their heritage (Taylor, 2007).
	Monitoring and enforcement	Socioeconomic development: rural livelihoods & well‐being	
		S‐SE2	Other detriments of law enforcement measures for rural communities
			Rhinoceros: Poaching in rural communities around Kruger National Park: ambiguity toward poaching; pros for local people: (financial) security, indirect benefits like basic roads, water wells, and so forth from rhinoceros poachers and kingpins, whereas the state and private/public partnerships have failed in achieving tangible benefits and a sense of security; (ii) cons especially for women: impaired social fabric of village life and cause for fear of losing relatives through law enforcement (Hübschle, 2017). Rhinoceros: Poachers compared with law enforcement officials in Mangalane (Mozambique): Poor community members (i) dislike poachers due to perceived threat to the community's social security and (ii) struggle with enforcement officials due to perceived antipoor enforcement of conservation‐prone, restrictive policies for protected area, despite a generally positive attitude toward the protected area (Vundla, 2019).Rhinoceros: Militarization of antipoaching in Mozambique's Limpopo National Park: villagers fear further violent antipoaching tactics of park officials and rhinoceros poachers, such as burning houses of villagers rightly or wrongly associated with poaching; villagers suspect reenvisioned resettlement strategy to achieve conservation outcomes (Witter & Satterfield, 2019).
Transactional	International restrictions	Socioeconomic development: national economies	
		T‐SE1	Trade bans' potential to significantly harm wildlife economies
			Elephant: International ivory trade ban of 1989: potential economic contribution of elephants in Botswana was effectively halved over a 15‐year period, as the ban eliminated certain direct‐use values of elephants (Barnes, 1996).

**TABLE 4:** Themes of demand‐side measures to restrict trade in iconic species, specifically elephants, rhinoceroses, lions, and pangolins, with corresponding research findings from evidence‐based impact studies illustrating the impact and effectiveness of governance measures targeted to restrict wildlife trade on demand in consumer countries with a focus on Angola, Botswana, Namibia, Malawi, Mozambique, South Africa, Tanzania, Zambia, and Zimbabwe.

		Output: Impact on	
	Input	Code	theme
		Demand in consumer countries (consumer sales volumes and prices)	
Demand‐side measures	Laws, policies, regulations, and associated campaigns	D‐D1	Potential of law enforcement coupled with government campaigns to reduce demand in consumer countries
			Elephant: Effective anti‐ivory campaigns in conjunction with the ivory trade ban in 1989 are associated with stigmatizing buying or possessing ivory in America, Europe, and Japan, reportedly reducing demand in these countries, resulting in smaller postban ivory markets throughout Africa, except for Nigeria (Stiles, 2004). Thailand limiting domestic ivory trade exclusively to products coming from registered captive Asian Elephants (*Elephas maximus*) in 2015 while banning the import, export, and trade in African Elephants (*Loxodonta africana*) resulted in a significant reduction of legal ivory trade in Bangkok, inferred from a downward trend in the number of shops selling ivory and the number of traded ivory products (Krishnasamy et al., 2016). Powerful campaigns of the Chinese government using Radio, Film, and Television showed effects in reducing extravagant consumption and developing negative attitudes toward buying ivory products of the younger generation, while the campaigns are thought to have contributed to a drop in the price of illegal ivory (Zhou et al., 2018). ‐ A pre‐ and postban survey in Chinese cities related to China's shutdown of all licensed ivory carving factories and retailers at the end of December 2017 indicated both a significantly grown proportion of ivory rejectors and a decline in ivory purchases, despite a persistent residuum of “Diehard Buyers” indicating that demand might not be completely preventable (Meijer et al., 2018). Rhinoceros: ‐ A generalized linear model of rhinoceros poaching in South Africa suggests that in addition to increasing antipoaching efforts and monetary fines upon conviction, governance improvements in Vietnam would be crucial to maintaining rhinoceros populations by helping to reduce poaching (Di Minin et al., 2015).

## RESULTS

**FIGURE 4:**
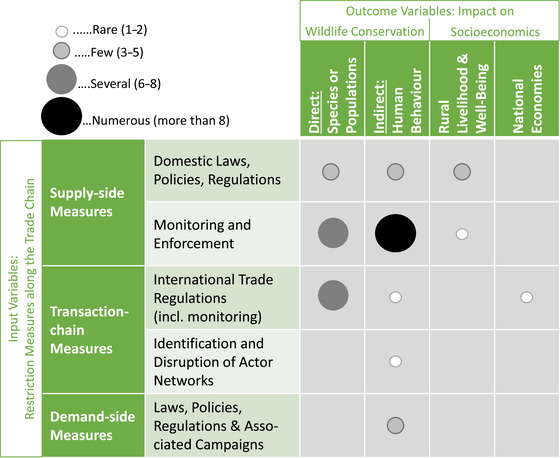
Frequency of studies based on the analytical framework to synthesize existing evidence on the impact and effectiveness of wildlife trade restrictions at reducing threats to iconic wildlife species in terms of wildlife conservation and socioeconomic outcomes in SADC countries (Angola, Botswana, Namibia, Malawi, Mozambique, South Africa, Tanzania, Zambia, and Zimbabwe).

The literature search and systematic review yielded 46 wildlife trade‐related impact studies relevant to the SADC region, focusing on the highlighted species. We found a strong focus on impact studies investigating supply‐side and transaction‐chain wildlife trade measures as input variables and the effects of monitoring and enforcing these measures as output variables (Figure 4) (references to corresponding studies with emergent thematic clusters in Appendices S3–S7).

The following main research areas emerged (Figure 4): law enforcement measures impacting human behavior (especially illegal hunting) or human attitudes toward wildlife crimes, particularly among rural communities (*n* = 20); the effects of law enforcement on wildlife conservation outcomes at the species or population level (*n* = 6); and the impact of international trade restrictions (in the form of CITES Appendix I listings) on wildlife conservation outcomes (*n* = 6). Furthermore, some research was conducted on the impact of legal supply restriction measures (including laws, policies, and regulations for spatial or species‐related constraints, such as quotas) on wildlife conservation (*n* = 7) and rural livelihoods (*n* = 3) and of demand‐side measures on conservation‐related behavior or attitudes (*n* = 5) (Figure 4).

The effects of the identification and disruption of actor networks and demand‐side measures were underresearched (Figure 4). Furthermore, none of the identified studies evaluated the impact of demand‐side legal measures to reduce the purchase, consumption, and possession of wild animals or wildlife products domestically (i.e., in SADC range states). However, few studies assessed the effectiveness of demand‐related restrictions in the target consumer countries, mainly in Southeast Asia. Further apparent gaps included a lack of research on the socioeconomic impacts of wildlife trade restrictions on rural livelihoods and national economies (Figure 4).

The following subsections summarize the themes emerging from our qualitative analysis of the identified impact studies (detailed thematic analysis in Tables 2, 3, 4). For the areas with the most obvious research gaps highlighted above, we briefly discuss the existing insights and evidence in the final 2 subsections (i.e., impact studies on rural livelihoods, well‐being, and national economies and insights into demand reduction for wildlife products of SADC origin).

### Impacts of international trade restrictions and related measures on reducing threats to species

International trade restrictions tend to be guided by CITES listings and include measures such as restrictions on cross‐border trade for primarily commercial purposes, permitting requirements, and quotas. Our qualitative analysis generated 4 themes in relation to international trade regulations. All themes relate to the effectiveness of CITES trade bans (i.e., Appendix I listings) (Table 2, T‐C1–T‐C4). (Theme codes in parentheses here in “Results” refer to summarized conservation‐related research findings in Table 2.) The themes highlighted the ambiguity and uncertainty of the effectiveness of such measures for wildlife conservation. The results of the few existing impact evaluations varied considerably in their assessment of the ecological, human behavioral, and economic effects at the macro level. This substantially compromised our ability to infer direct links relating to the impact of international‐level regulations.

Impact studies on rhinoceros and pangolin species as well as on lions confirmed concerns that some international trade bans may simply drive markets underground instead of exerting a discernible positive influence on species populations (theme code T‐C1 [Table 1]) (Conrad, 2012). Furthermore, evidence‐based research on African elephants suggested that unregulated markets, widespread corruption, civil unrest, or poor wildlife management increase the likelihood of rendering trade bans ineffective (T‐C2). These findings need to be considered in the light that some countries might rather need to address an elephant overpopulation challenge. An early economic model based on data from Zambia reflected that optimal elephant stock was consistently lower than the estimates of elephant populations for both trade‐ and no‐trade scenarios, suggesting that such countries might need to reduce those levels actively (through culling) or passively (through underinvestment in protection) (Bulte & van Kooten, 1999).

We found examples of short‐term trade bans benefitting conservation in conjunction with further conditions (T‐C3). However, we noted a persistent debate over the role of post‐ban efforts to enable subsequent use and trade, based on cases lauded as trade‐ban successes, notably crocodilians (*Crocodilia*) and the vicuňa (*Vicugna vicugna*), for which CITES coupled strict trade restrictions with efforts to support nations in establishing sustainable use programs (Lichtenstein, 2010; Moyle, 2013; Thorbjarnarson, 1999). One theoretical model suggested a trade ban was likely to positively affect elephant populations if it achieved the following: a widespread moral demand‐reducing effect; improved interception of smuggled products; limited increases of ivory stockpiles from official production; and no reduction in law enforcement efforts (Heltberg, 2001). Another theoretical model indicated that a successful trade ban would need to deal effectively with the so‐called free‐rider problem, whereby nations placing a high existence value on elephants without actively investing in their survival are classified as free riders (Khanna & Harford, 1996). The authors argue that an international transfer payment mechanism would support conservation strategies in the producer countries.

Intelligence‐led, proactive interruption and inhibition of illegal hunting activities were regularly emphasized as measures to render international trade restrictions effective (Hübschle, 2016). However, corresponding evidence‐based impact studies were few. The only emerging theme from the qualitative analysis was a lack of collaborative approaches across jurisdictions (T‐C4). As a result, very few large‐scale seizures prompted successful investigations or arrests and almost no convictions. Research suggested that failure to disrupt criminal networks more effectively may result from missing federated criminal evidence data and trust as a basis for legal prosecuting provisions between different jurisdictions (Haas & Ferreira, 2015; Nanima, 2019); a lack of complements, such as scanners, X‐ray devices, sniffer dogs, and so forth (Milliken, 2014); a lack of coordinated intelligence activities; and inadequate legislation to prosecute individuals (Hübschle, 2017; Nanima, 2019).

### Impacts of domestic trade restrictions on reducing threats to species

Studies investigating the impact of domestic laws, policies, and regulations to restrict wildlife harvesting and supply on wildlife conservation outcomes elicited 3 synthesized themes (Table 2, S‐C1–S‐C3). Wildlife hunting bans, moratoria, and quotas constituted the typical domestic restriction measures in the identified impact studies. Studies of hunting prohibitions on lions and elephants suggested that these measures only benefited strained animal populations in the short term (S‐C1). The associated loss of livelihood and human well‐being for rural communities during the hunting suspension led to uncontrolled meat poaching and human encroachment and an even greater risk for wild animal populations in the longer term. A second theme indicated detrimental consequences for social animal structures, such as elephants, when hunting quotas across national borders were uncoordinated (S‐C2). Another theme suggested that restrictions were more effective at modulating human behavior in favor of wildlife conservation when framed as permissions rather than prohibitions (S‐C3).

### Impacts of domestic monitoring and enforcement on reducing threats to species

Most evidence‐based research on dedicated trade‐restriction measures examined the impact of efforts to monitor and enforce trade‐restrictive laws, policies, and regulations on conservation‐related outcomes (Figure 2). Studies on measures such as patrol efforts, sentencing strategies, or intelligence elicited 5 corresponding themes (Table 2, S‐C4–S‐C8).

Numerous studies suggested that sufficient high‐quality and proactive law enforcement efforts are beneficial for wildlife conservation, both directly (on species or population level) and indirectly (on human behavior level) (S‐C4). In other words, impact studies showed positive effects of law enforcement measures to combat wildlife crime or achieve species conservation when these efforts were well‐resourced both quantitatively, in terms of human and financial capacities, and qualitatively, in terms of the capabilities of field rangers and leadership, even in cases when illegal hunting and fishing soared in countries experiencing economic hardship, such as in Zimbabwe after its economic collapse (Lindsey et al., 2011). Approaching monitoring and enforcement in a proactive rather than a reactive way (i.e., a shift from patrolling efforts to intelligence‐based approaches) was also found to positively influence conservation outcomes. Conversely, this theme included findings that poaching remained a constant and severe problem in cases of insufficient or incapable resources (S‐C4). The effectiveness of law enforcement efforts was sometimes measured in terms of discovering illegal activities ex post when the lives of targeted animals had already been lost, such as in Milledge (2007) who reported a 42% rhinoceros horn recovery rate through measures ranging from rapid response in protected areas to investigations and confiscations in urban centers in 9 African countries.

The research also showed that effective law enforcement hinges more on detection risk than on harm or loss (S‐C5). In other words, although measures to increase detection risk, such as patrol efforts, showed positive impacts on conservation outcomes, the potential harm or loss in case of detection (through prison sentences, fines, or other forms of financial loss) did not constitute a comparable deterrent, unless such penalties were raised to extreme levels, such as a shoot‐on‐sight policy for poachers. Furthermore, large‐scale prosecutions for wildlife crimes tended to be rare globally (Nowell, 2012). A third theme emerged, suggesting that new technologies show promise to enhance detection risk for illegal hunters (S‐C6). However, evidence‐based studies investigating the impact of these technological developments on conservation outcomes were rare (Figure 2). Most relevant studies were confined to field tests of various tech solutions under experimental conditions (Pimm et al., 2015).

Several authors argued that measures to change human behavior and attitudes for wildlife conservation purposes must couple law enforcement measures with other efforts to mitigate negative consequences for socioeconomic development (S‐C7). The identified impact studies suggested that such measures might need to address consumer demand, corruption, and poverty and be supplemented by regular monitoring of animal populations. The final theme linked the effectiveness of law enforcement to nonmarket‐related social constructs of a society's relationship with an animal species (S‐C8). Evidence‐based investigations highlighted the significance of social legitimacy factors in supporting or undermining domestic law enforcement efforts (Hübschle, 2017).

### Impact of trade restrictions on socioeconomic development (rural livelihoods and national economies)

The synthesized themes relating to this topic are summarized in Table 3 (with theme codes appearing below in parentheses). We found evidence‐based impact studies of restrictive measures along the trade chain on socioeconomic development outcome variables were sparse in comparison with conservation‐related outcomes (Figure 2). Consequently, the qualitative thematic analysis of findings was limited to a small number of studies, even though such impacts could plausibly be significant and lead to adverse secondary conservation outcomes.

Our analyses elicited the potential of trade bans to significantly harm wildlife economies as the only theme for the socioeconomic impact of international trade restrictions (T‐SE1). Any effects of measures restricting trade activities on rural livelihoods were researched on a domestic level. Domestically, hunting restrictions overwhelmingly demonstrated adverse impacts on rural livelihoods and well‐being (S‐SE1). Imposed costs included lost food sources, assets, jobs, or other forms of income, thereby aggravating already poverty‐stricken areas. This theme was supported by other research emphasizing the complementary benefits of both tourism and hunting for rural communities in Namibia because the benefits occurred at different times, appeared in different places, and reached different sections of local communities (Naidoo et al., 2016). Similarly, a survey on the effects of the US suspension on imports of captive lion trophies from South Africa highlighted alleged job losses in rural areas, thereby indicating the impact of trade restrictive measures on livelihood outcomes (Williams & ’t Sas‐Rolfes, 2019).

In addition to material hardship, researchers identified how law enforcement measures can result in other detriments for rural communities, such as a loss of the social fabric in a village (S‐SE2). The studies in this theme highlighted how rural communities experience both benefits and costs through illegal hunting, especially when the state failed to deliver basic public services in poverty‐stricken areas (S‐SE2). However, this research also highlighted the ambiguous relationships of rural communities with poachers and law enforcement officers. Although poachers can be seen to threaten secure village life, there was also evidence of adverse treatment of villagers by law enforcement staff and associated fear of violent antipoaching tactics, despite the fact that entire villages are not necessarily involved in or complicit with illegal hunting of high‐value species. In their most extreme form, conservation‐related enforcement measures were reportedly used as a means for renewed dispossession and displacement of rural communities (Massé & Lunstrum, 2016).

### Consumer demand reduction

Our review did not elicit evidence‐based research studies assessing direct links between demand‐side measures to restrict trade‐related activities and their effect on wildlife conservation or socioeconomic development in SADC range states (Figure 2; Table 4). Reductions in consumer demand were often presented as a proxy outcome variable to infer themes for reduced threats to species by means of legal measures to restrict activities along the trade chain. However, consumer demand reduction as an outcome variable was challenging to ascertain, partly because illegal purchases and consumption are clandestine and partly because demand is multidimensional and difficult to measure by a single attribute. Both sale volumes (quantities of goods sold per unit of time) and product prices would be relevant, especially the latter. Due to the ambiguity of the net effects of declining volumes accompanied by increasing prices, depending on the price elasticity of demand (’t Sas‐Rolfes, 2012), the only certain measure of declining demand would have been a confirmed simultaneous decline in both consumer sales volumes and prices.

Coupling law enforcement with consumer‐targeted government campaigns emerged as the one theme to enhance the potential of reducing demand in consumer countries (with a focus on ivory) (D‐D1). The relevant studies emphasized the potency of combining trade bans with enforcement measures and consumer campaigns to stigmatize the purchase or possession of ivory to effectively reduce demand. However, some researchers suggested that media campaigns and changing fashion alone provide a plausible alternative explanation for reversing the decline in Africa's elephant population (Challender et al., 2015). Overall, the full impact of regulatory interventions in China in late 2011 remained somewhat unclear (Gao & Clark, 2014), as did Vietnam's penal code revisions. The evidence for successful demand reduction of other relevant species products was less compelling. Although the domestic trade restrictions in Asian rhinoceros horn consumer countries in the early 1990s are believed to have been effective, the subsequent obvious surge in East Asian consumer prices in the early 21st century suggested that the effects were, in part, only temporary (’t Sas‐Rolfes, 2012).

To support regulation, NGOs made attempts at voluntary consumer demand reduction. These attempts applied a range of techniques, from public awareness campaigns and celebrity endorsements to more targeted social marketing techniques. Independent assessments of these attempts pointed to various and significant shortcomings (Dang Vu et al., 2020; Olmedo et al., 2020, 2018; Veríssimo & Wan, 2019), and their ultimate impact to date remains uncertain. There have been relatively few demand reduction interventions grounded in appropriately structured consumer research guided by behavioral science and even fewer robust impact evaluations of such interventions (UNEP, 2019).

## DISCUSSION

The role of trade in relation to other factors driving wildlife species decline remains a somewhat uncertain and contested issue. The relatively low number (46) of studies that constituted acceptable evidence‐based evaluations related to this question was striking. Although wildlife trade has recently gained further significant prominence as both a perceived threat and a catalyst for conservation activism (’t Sas‐Rolfes et al., 2019), many assessments of species decline pointed to habitat loss and fragmentation as the most salient long‐term drivers. At the time of the 1989 CITES ivory trade ban, economists contemplated whether it would serve as a long‐term solution for African elephant conservation and concluded that it would not (Barbier et al., 1990). Around the same time, Wilson (1989) highlighted the main factor driving 20th‐century biodiversity loss as anthropogenic habitat destruction, fueled by the rapid growth of the global human population and associated economic activity.

In a subsequent analysis, Swanson (1994) argued that trade restrictions are most appropriate for genuine open‐access resources (e.g., marine species) because they provide a deterrent effect to slow down the rate of harvesting. However, for terrestrial species that occupy land with other potential uses and require the active investment of resources to manage and protect them, the associated opportunity costs with trade restrictions can undermine the long‐term economic case for conserving such species. If illegal consumer markets for their harvested products persist, they become increasingly costly to protect from commercial poaching. They may eventually be viewed as economic liabilities rather than assets, especially in the case of species that pose potential threats to the lives and livelihoods of local people. In such instances, reestablishing controlled and sustainably supplied legal trading regimes may be a better long‐term option to ensure species persistence.

Following a broader dialogue with inputs from both ecologists and economists, Swanson (1995) noted deep ideological differences over whether biodiversity conservation is best achieved through shielding nature from human development or through human development, which may also influence the perceptions of both relative threats to wildlife and the most appropriate measures to address these. Such differing emphases were reflected in recent global assessments of species threats. For example, one claimed that overexploitation is the most prevalent threat currently facing species (Maxwell et al., 2016), whereas another identified habitat loss as the more salient factor (Tilman et al., 2017). Nevertheless, it is notable that of the 11 categories of the IUCN Red List's current threat classification system, only 2—biological resource use and spread of invasive and problem species, pathogens, and genes—are (indirectly) associated with wildlife trade.

The IUCN Red List assessments identified illegal harvesting for international trade as the central threat to the 2 rhinoceros species (Emslie, 2020; Emslie et al., 2016) and a growing threat to pangolins, which are also harvested for domestic consumption and trade and threatened by habitat loss (Nixon et al., 2019; Pietersen et al., 2019). However, the African elephant assessment emphasized that although illegal hunting for ivory and meat remains a key factor in specific areas, the most important perceived threat is habitat loss and fragmentation, aggravated by increasing human–elephant conflict (Gobush et al., 2021). The lion assessment listed indiscriminate killing and prey depletion, accompanied by habitat loss and fragmentation, as key factors, with a minor role of poorly managed trophy hunting in some instances (Bauer et al., 2016). It noted only a small role for domestic trade and some concern over a potential future international trade in lion body parts.

The SADC region currently harbors 95% of Africa's rhinoceroses, 80% of its elephants, and a similarly high proportion of its wild lions. Given the goal of conserving viable free‐ranging populations of these species and others, such as pangolins, and given that they are all at least to some extent threatened by illegal and unsustainable harvesting for trade purposes, how is this threat best addressed? What options and strategies might improve the effectiveness of international, regional, and national wildlife trade regulatory mechanisms to sustainably disrupt the illegal wildlife trade in the region? The existing literature offered some guidance on this but also exposed some glaring research gaps and uncertainties and raised questions about whether certain current trade‐related policies provide the most effective long‐term strategy for species conservation.

Our review results indicated that CITES listings, when viewed as a stand‐alone measure, offered limited protection to these species, constrained by varying perceptions of social legitimacy among market actors and with potentially even detrimental conservation and socioeconomic outcomes. Announcements of unconditional or wide‐ranging trade restrictions, such as export or import bans, might even abruptly increase species threats by signaling scarcity in the marketplace, resulting in higher prices and increased incentives for illegal activity, which may persist (Hall et al., 2008; Rivalan et al., 2007). Our results suggested that, to be effective, CITES listings must be supported by a fully aligned range of appropriate measures in range, transit, and consumer countries. The CITES trade restrictions for rhinoceros, elephant, pangolin, and lion alike supported this insight that stand‐alone Appendix I listings tend to be ineffective in the longer term.

The 1977 CITES Appendix I rhinoceros listings failed to prevent the further poaching and decline of Africa's black rhinoceros. At the continental level, populations only started to recover after 4 key Asian consumer countries agreed to take measures to outlaw their domestic markets in 1993, following direct diplomatic pressure from the United States. Even so, under complete ban conditions, rhinoceros poaching resurged significantly from 2007 onward, not only slowing and threatening the recovery of black rhinoceros populations but also starting to reverse the previously impressive southern white rhinoceros (*Ceratotherium simum simum*) recovery. Uninterrupted since the 1960s, the latter recovery took place with the support of some legal trade measures for live animals and hunting trophies (’t Sas‐Rolfes et al., 2022). Importantly, the Appendix I listings also failed to prevent the loss of 2 subspecies, the northern white rhinoceros (*Ceratotherium simum cottoni*) and the northwestern black rhinoceros (*Diceros bicornis longipes*) (neither of which occurred in the SADC region).

Despite a reasonable body of research on the topic, the role of CITES in protecting African elephants continues to be disputed. Although it is widely agreed that the 1989 Appendix I listing suppressed key consumer markets and slowed the rate of poaching at the time, it is also clear that the subsequent benefits of the ivory ban under the CITES regime have been unevenly distributed and that local law enforcement efforts remain critical (Jachmann & Billiouw, 1997). Certain countries with adequately protected and sizable populations carry a disproportionately high burden in the form of management and opportunity costs. Attempts to address this imbalance by way of split listing (i.e., downlisting populations of well‐performing countries to Appendix II and then allowing one‐off ivory sales) have created confusion and tension. Critics blamed these on resurgent consumer demand in the early 21st century (’t Sas‐Rolfes et al., 2014). As a result, no further sales are currently contemplated, and ivory trade policy continues to be fiercely debated. However, recent analyses confirmed the importance of other domestic factors (e.g., poverty and corruption) in influencing elephant conservation performance (Kuiper et al., 2023). Furthermore, a comprehensive study of historical ivory prices (Do et al., 2021) suggested that elephant poaching is relatively inelastic. This implies that the overall effect of the CITES ban itself is relatively neutral, neither encouraging nor discouraging poaching significantly. This result appears to be supported by another recent study that showed fairly persistent poaching levels, especially in the southern African region (Schlossberg et al., 2020).

The effect of the recent CITES Appendix I listing of pangolins is very difficult to determine, as wild population numbers are challenging to monitor. Nevertheless, early signs are not encouraging. Since the uplisting, substantially higher seizure levels have been reported, suggesting that illegal exploitation persists at worrying levels. The effect of the 2016 CITES decision to restrict lion trade (apart from trophies) to the export of body parts from captive‐bred animals in South Africa, subject to a quota restriction, is the subject of ongoing investigations. However, as with elephants, a confusing inconsistency of policy direction amid fiercely contested views confounds attempts to analyze this issue with neutrality and reach conclusive results. Nonetheless, there was no substantial evidence that the lion body part trade is driving significant levels of poaching at the regional level.

The socioeconomic impacts of CITES listings at national and, especially, local community levels were inadequately researched, and the opportunity costs imposed on the countries with good conservation track records that oppose them are potentially substantial. This is a cause for concern, especially given the current lack of long‐term vision for elephant ivory and rhinoceros horn trade. Both will continue to accumulate in collected stockpiles as long as populations of elephants and rhinoceroses continue to survive and naturally produce these harvestable and storable products. Furthermore, with evidence of persistent residual demand in consumer countries despite domestic trade bans, all indications were that these species will require the continued allocation of significant resources to protect them from poaching. Given the budgetary constraints facing many SADC governments, likely aggravated by the economic impacts of the COVID‐19 pandemic, it is unclear whether these resources will be available at adequate levels. Consistent reports of severe shortfalls in conservation funding suggest that there will be an ongoing, if not increasing, dependence on external donor support, which may be insufficient and insecure (Lindsey et al., 2018; McCarthy et al., 2012). Furthermore, significant proportions of the SADC populations of elephants, rhinoceroses, and lions are found on working lands rather than protected areas and may be replaced by domestic species or other forms of land use if they represent a socioeconomic burden.

Research on the impacts of domestic restrictions, such as trophy hunting moratoria, confirms that these can have conservation‐positive impacts in the short term, allowing affected species populations to recover in certain state or communal areas. However, they also typically have negative socioeconomic impacts at local levels, which can stimulate illegal activity in response and lead to adverse conservation impacts in the longer run. The research suggested that harvesting should be re‐instated once populations recover under better‐managed conditions with appropriate incentives (such as the bonus–malus system for lion trophy hunts adopted in Mozambique). It also suggested that domestic measures should be coordinated across national boundaries in transfrontier conservation areas. It further indicated that certain domestic restrictions may be undermined by a lack of social legitimacy (e.g., the rhinoceros horn trade ban in South Africa and Mozambique). Research on the impacts of domestic restrictions (especially those linked to substantial illegal activity) on national economies was noticeably lacking.

The extensive body of research on domestic monitoring and enforcement confirmed that positive site‐specific results are correlated with adequate funding but reaffirms the significance of social legitimacy as a supporting or undermining factor. The research also pointed to a high probability of early detection and interception of protected area incursions and other illegal activity as being the most effective deterrent with the highest positive conservation impact. Our results suggested that although increasing the severity of penalties has a positive effect, this is substantially undermined if the probability of apprehension and actual punishment is low. Notwithstanding the growing suite of technologies to support monitoring and antipoaching measures, the ultimate impact of these remained largely unknown and needs to be evaluated relative to their costs and other implementation feasibility factors to assess potential scalability.

Although measures to support trade restrictions, such as the disruption of criminal networks and efforts aimed at demand reduction, were widely considered imperative, the evidence‐based research on their effectiveness to date was limited and not especially encouraging for the species of regional concern. Disruption of transnational criminal networks was considered necessary to discourage illegal activity relating to smuggling high‐value goods such as rhinoceros horn, ivory, pangolin scales, and lion fangs. However, there was no substantial evidence of positive conservation impacts to date or to suggest that disruption can prevent these smuggling activities altogether, especially when sophisticated networks undertake these with compliance from corrupt officials and links to other lucrative criminal enterprises involving, for example, timber, arms, and drugs. Similarly, although review results on measures to restrict demand for ivory suggested potential to reduce demand in consumer countries when coupled with government campaigns, this did not appear to have translated into markedly reduced elephant poaching rates, and tangible impacts on consumer demand for rhinoceros horn were less clear. The evidence suggested that such efforts may somewhat reduce demand but not eliminate it entirely, leaving the question of how best to tackle the persistent residual consumers.

The experience to date with demand reduction revealed a potential flaw with past approaches, which have tended to outlaw product purchases first and then attempted to engage with consumers in clandestine illegal market environments. Future attempts at demand reduction might benefit from consumer engagement and associated attempts at voluntary behavior change measures in legal markets before implementing more coercive restrictions. Future attempts should also be subject to design informed by behavioral science insights and subject to robust impact evaluations.

Whether permanent bans or legal trading regimes are the best long‐term option for various species remains a highly contested and poorly understood issue, necessitating further neutral investigation by appropriately qualified analysts. Given the findings of Do et al. (2021), it seems likely that, at least for elephants, there can be no clear answers. Conservation success will variably depend largely on other local factors, regardless of whether legal trade takes place or not (unless legalizing trade creates direct and meaningful local benefits). For other species, if legal trade is to succeed as a conservation measure, it will likely depend on creating bespoke institutional arrangements (e.g., strong property rights and suitably competitive market mechanisms) with appropriate control measures, such as single‐channel marketing systems, accompanied by source certification and traceability measures to support enforcement and deter laundering of illegal products. Supporting technologies for such approaches have already been developed, such as the RhODIS DNA tracking system for rhinoceroses (Harper et al., 2018), and further research is being conducted to assess the effectiveness of chain‐of‐custody systems for lion bone exports from South Africa (Williams et al., 2021). However, for SADC states that may benefit from legal trade to win over others that may not, further investigation of supporting measures and other necessary assurances will be required. Such research should include likely consumer demand responses, following the example of Hanley et al. (2018), who conducted choice modeling of rhinoceros horn demand in Vietnam and identified preferences for humanely harvested wild horn from the least rare species.

Although several of southern Africa's iconic species are threatened to various extents by excessive illegal exploitation for trade purposes, this threat must be assessed within the broader context of socioecological factors driving regional biodiversity decline. Although this threat is dominant for rhinoceros species in particular, wild mammal populations are more substantially threatened by the broader forces of an expanding human footprint. Forecasts suggest that, especially in the northern parts of the region, projected human population growth and associated economic development imperatives will place substantial additional pressure on land and natural resources, compounding the existing habitat conversion and fragmentation problem that threatens free‐ranging populations of large mammals such as elephants and lions. Robust conservation approaches must adopt strategies to enable these species to move freely between certain protected areas across communal and private lands to mitigate the effects of habitat fragmentation.

Species with valuable harvestable products may be threatened by poaching incursions into protected areas; outside of these publicly funded areas (i.e., on privately funded working lands), such species are most likely to survive in situations where they provide meaningful net benefits to landholders. This suggests that, in the context of developing SADC economies, realistic achievement of species conservation outside of protected areas must embrace inclusive and innovative wildlife management practices to achieve human development goals. In other words, although protected area managers may be mandated to shield conservation from development, this luxury is largely absent outside these areas. In a post‐COVID‐19 world economy, these pressures are only likely to increase (Lindsey et al., 2020; Roe et al., 2020).

Our review yielded implications for future policy and revealed research gaps that deserve attention. Inside protected areas, early detection and apprehension of incursions remain critical and local enforcement capacity should be developed according to principles of situational crime prevention. Outside of protected areas, constructive engagement with relevant local communities is critical. In general, the regional socioeconomic contribution of wildlife remains inadequately researched and understood at all levels and deserves more attention, from local human benefit flows and associated conservation incentives to broader contributions to national economies and global society. Finally, there is an ongoing need to research and identify innovative sources of sustainable finance for conservation to fill the yawning funding gap, including through the possible development of sustainable wildlife product markets. Unconditional and wide‐ranging trade restrictions—such as CITES Appendix I listings—have not demonstrated unqualified success for the reviewed species cases, and more nuanced approaches seem justified.

## Supporting information

Appendix S1: List of databases and organizations for grey literature search, including successful search terms per website
Appendix S2: Review and data extraction protocol applied to eligible studies after applying the selection criteria

## References

[cobi14262-bib-0001] Abensperg‐Traun, M. (2009). CITES, sustainable use of wild species and incentive‐driven conservation in developing countries, with an emphasis on southern Africa. Biological Conservation, 142(5), 948–963.

[cobi14262-bib-0002] Ball, M. B. , Wenham, C. M. , Clegg, B. W. , & Clegg, S. B. (2018). What does it take to curtail rhino poaching? Lessons learned from twenty years of experience at Malilangwe Wildlife Reserve, Zimbabwe. Pachyderm, 60, 96–104.

[cobi14262-bib-0003] Barbier, E. B. , Burgess, J. C. , Swanson, T. M. , & Pearce, D. W. (1990). Elephants, economics and ivory (1st ed.). Earthscan Publications.

[cobi14262-bib-0004] Barichievy, C. , Munro, L. , Clinning, G. , Whittington‐Jones, B. , & Masterson, G. (2017). Do armed field‐rangers deter rhino poachers? An empirical analysis. Biological Conservation, 209, 554–560.

[cobi14262-bib-0005] Barnes, J. I. (1996). Changes in the economic use value of elephant in Botswana: The effect of international trade prohibition. Ecological Economics, 18(3), 215–230.

[cobi14262-bib-0006] Bauer, H. , Chapron, G. , Nowell, K. , Henschel, P. , Funston, P. , Hunter, L. T. B. , Macdonald, D. W. , & Packer, C. (2015). Lion (*Panthera leo*) populations are declining rapidly across Africa, except in intensively managed areas. Proceedings of the National Academy of Sciences of the United States of America, 112(48), 14894–14899. 26504235 10.1073/pnas.1500664112 PMC4672814

[cobi14262-bib-0007] Bauer, H. , Packer, C. , Funston, P. F. , Henschel, P. , & Nowell, K. (2016). Panthera leo . The IUCN Red List of Threatened Species 2016. 10.2305/IUCN.UK.2016-3.RLTS.T15951A107265605.en

[cobi14262-bib-0008] Baylis, K. , Honey‐Rosés, J. , Börner, J. , Corbera, E. , Ezzine‐de‐Blas, D. , Ferraro, P. J. , Lapeyre, R. , Persson, U. M. , Pfaff, A. , & Wunder, S. (2016). Mainstreaming impact evaluation in nature conservation. Conservation Letters, 9(1), 58–64.

[cobi14262-bib-0009] Begg, C. M. , Miller, J. R. B. , & Begg, K. S. (2018). Effective implementation of age restrictions increases selectivity of sport hunting of the African lion. Journal of Applied Ecology, 55(1), 139–146.

[cobi14262-bib-0010] Beinart, W. , & Coates, P. (2002). Environment and history: The taming of nature in the USA and South Africa. Routledge. 10.4324/9780203133552

[cobi14262-bib-0011] Bondi, E. , Fang, F. , Hamilton, M. , Kar, D. , Dmello, D. , Choi, J. , Hannaford, R. , Iyer, A. , Joppa, L. , Tambe, M. , & Nevatia, R. (2018). SPOT poachers in action: Augmenting conservation drones with automatic detection in near real time . Proceedings of the Thirty‐Second AAAI Conference on Artificial Intelligence, New Orleans, LA, USA, 2–7 February 2018; pp. 7741–7746.

[cobi14262-bib-0012] Braun, V. , & Clarke, V. (2006). Using thematic analysis in psychology. Qualitative Research in Psychology, 3, 77–101.

[cobi14262-bib-0013] Bulte, E. H. , & van Kooten, G. C. (1999). Economics of antipoaching enforcement and the ivory trade ban. American Journal of Agricultural Economics, 81(2), 453–466.

[cobi14262-bib-0014] Burn, R. W. , Underwood, F. M. , & Blanc, J. (2011). Global trends and factors associated with the illegal killing of elephants: A hierarchical Bayesian analysis of carcass encounter data. PLoS ONE, 6(9), Article e24165. 21912670 10.1371/journal.pone.0024165 PMC3166301

[cobi14262-bib-0015] Challender, D. W. S. , Harrop, S. R. , & MacMillan, D. C. (2015). Understanding markets to conserve trade‐threatened species in CITES. Biological Conservation, 187, 249–259.

[cobi14262-bib-0016] Cheng, S. H. , Robinson, J. E. , Cox, N. , Biggs, D. , Olsson, A. , Mascia, M. B. , & McKinnon, M. C. (2017). Mapping the evidence: Effectiveness of international wildlife trade practices and policies. Conservation International. https://www.researchgate.net/publication/318725745

[cobi14262-bib-0017] Cheteni, P. (2014). An analysis of antipoaching techniques in Africa: A case of rhino poaching. Environmental Economics, 5(3), 63–70.

[cobi14262-bib-0018] CITES Secretariat . (2007). SC55 Doc. 10.2 (Rev. 1): Interpretation and Implementation of the Convention Species: Species trade and conservation issues: Elephants: Mike Baseline Info. CITES.

[cobi14262-bib-0019] Conrad, K. (2012). Trade bans: A perfect storm for poaching? Tropical Conservation Science, 5(3), 245–254.

[cobi14262-bib-0020] Dang Vu, H. N. , Nielsen, M. R. , & Jacobsen, J. B. (2020). Reference group influences and campaign exposure effects on rhino horn demand: Qualitative insights from Vietnam. People and Nature, 2(4), 923–939.

[cobi14262-bib-0021] Di Minin, E. , Laitila, J. , Montesino‐Pouzols, F. , Leader‐Williams, N. , Slotow, R. , Goodman, P. S. , Conway, A. J. , & Moilanen, A. (2015). Identification of policies for a sustainable legal trade in rhinoceros horn based on population projection and socioeconomic models. Conservation Biology, 29(2), 545–555. 25331485 10.1111/cobi.12412 PMC4405060

[cobi14262-bib-0022] Do, Q.‐T. , Levchenko, A. A. , Ma, L. , Blanc, J. , Dublin, H. , & Milliken, T. (2021). The price elasticity of African elephant poaching. The World Bank Economic Review, 35(3), 545–562.

[cobi14262-bib-0023] Drury, R. , Homewood, K. , & Randall, S. (2011). Less is more: The potential of qualitative approaches in conservation research. Animal Conservation, 14(1), 18–24.

[cobi14262-bib-0024] Emslie, R. (2020). Diceros bicornis . The IUCN Red List of Threatened Species 2020. 10.2305/IUCN.UK.2020-1.RLTS.T6557A152728945.en

[cobi14262-bib-0025] Emslie, R. , Adcock, K. , Emslie, R. H. , Milliken, T. , Talukdar, B. , Ellis, S. , & Knight, M. H. (2016). African and Asian Rhinoceroses—Status, Conservation and Trade A report from the IUCN Species Survival Commission (IUCN SSC) African and Asian Rhino Specialist Groups and TRAFFIC to the CITES Secretariat pursuant to Resolution Conf. 9.14 (Rev. CoP15) . IUCN SSC African Rhino Specialist Group and CITES.

[cobi14262-bib-0026] Ferreira, S. M. , Bissett, C. , Cowell, C. R. , Gaylard, A. , Greaver, C. , Hayes, J. , Hofmeyr, M. , Moolman‐van der Vyver, L. , & Zimmermann, D. (2017). The status of rhinoceroses in South African national parks. Koedoe, 59(1), Article 1392. 10.4102/koedoe.v59i1.1392

[cobi14262-bib-0308] Ferreira, S. M. , Greaver, C. , Nhleko, Z. , & Simms, C. (2018). Realization of poaching effects on rhinoceroses in KrugerNational Park, South Africa. African Journal of Wildlife Research, 48(1), Article 013001.

[cobi14262-bib-0027] Galvin, K. A. , Beeton, T. A. , & Luizza, M. W. (2018). African community‐based conservation: A systematic review of social and ecological outcomes. Ecology and Society, 23(3), Article 39. 10.5751/ES-10217-230339

[cobi14262-bib-0028] Gandiwa, E. , Heitkönig, I. M. A. , Lokhorst, A. M. , Prins, H. H. T. , & Leeuwis, C. (2013). Illegal hunting and law enforcement during a period of economic decline in Zimbabwe: A case study of northern Gonarezhou National Park and adjacent areas. Journal for Nature Conservation, 21, 133–142.

[cobi14262-bib-0029] Gao, Y. , & Clark, S. G. (2014). Elephant ivory trade in China: Trends and drivers. Biological Conservation, 180, 23–30.

[cobi14262-bib-0030] Gaodirelwe, I. , Masunga, G. S. , & Motsholapheko, M. R. (2020). Community‐based natural resource management: A promising strategy for reducing subsistence poaching around protected areas, northern Botswana. Environment, Development and Sustainability, 22, 2269–2287.

[cobi14262-bib-0031] Gaodirelwe, I. , Motsholapheko, M. R. , & Masunga, G. S. (2020). Community perceptions of wildlife management strategies and subsistence poaching in the Okavango Delta, Botswana. Human Dimensions of Wildlife, 25(3), 232–249.

[cobi14262-bib-0032] Gioia, D. A. , Corley, K. G. , & Hamilton, A. L. (2013). Seeking qualitative rigor in inductive research: Notes on the Gioia methodology. Organizational Research Methods, 16(1), 15–31.

[cobi14262-bib-0033] Gobush, K. S. , Edwards, C. T. T. , Balfour, D. , Wittemyer, G. , Maisels, F. , & Taylor, R. D. (2022). Loxodonta africana . The IUCN Red List of Threatened Species 2021. 10.2305/IUCN.UK.2021-1.RLTS.T181008073A181022663.en

[cobi14262-bib-0034] Haas, T. C. , & Ferreira, S. M. (2015). Federated databases and actionable intelligence: Using social network analysis to disrupt transnational wildlife trafficking criminal networks. Security Informatics, 4, Article 2. 10.1186/s13388-015-0018-8

[cobi14262-bib-0035] Hall, R. J. , Milner‐Gulland, E. J. , & Courchamp, F. (2008). Endangering the endangered: The effects of perceived rarity on species exploitation. Conservation Letters, 1(2), 75–81.

[cobi14262-bib-0036] Hanley, N. , Sheremet, O. , Bozzola, M. , & MacMillan, D. C. (2018). The allure of the illegal: Choice modeling of rhino horn demand in Vietnam. Conservation Letters, 11(3), Article e12417.

[cobi14262-bib-0037] Harper, C. , Ludwig, A. , Clarke, A. , Makgopela, K. , Yurchenko, A. , Guthrie, A. , Dobrynin, P. , Tamazian, G. , Emslie, R. , van Heerden, M. , Hofmeyr, M. , Potter, R. , Roets, J. , Beytell, P. , Otiende, M. , Kariuki, L. , du Toit, R. , Anderson, N. , Okori, J. , … O'Brien, S. J. (2018). Robust forensic matching of confiscated horns to individual poached African rhinoceros. Current Biology, 28(1), R13–R14. 29316411 10.1016/j.cub.2017.11.005

[cobi14262-bib-0038] Hart, A. G. , Rolfe, R. N. , Dandy, S. , Stubbs, H. , MacTavish, D. , MacTavish, L. , & Goodenough, A. E. (2015). Can handheld thermal imaging technology improve detection of poachers in African bushveldt? PLoS ONE, 10(6), Article e0131584. 26110865 10.1371/journal.pone.0131584 PMC4481516

[cobi14262-bib-0039] Hauenstein, S. , Kshatriya, M. , Blanc, J. , Dormann, C. F. , & Beale, C. M. (2019). African elephant poaching rates correlate with local poverty, national corruption and global ivory price. Nature Communications, 10(1), Article 2242. 10.1038/s41467-019-09993-2 PMC6538616 31138804

[cobi14262-bib-0040] Heinrich, S. , Wittmann, T. A. , Prowse, T. A. A. , Ross, J. v. , Delean, S. , Shepherd, C. R. , & Cassey, P. (2016). Where did all the pangolins go? International CITES trade in pangolin species. Global Ecology and Conservation, 8, 241–253.

[cobi14262-bib-0041] Heltberg, R. (2001). Impact of the ivory trade ban on poaching incentives: A numerical example. Ecological Economics, 36(2), 189–195.

[cobi14262-bib-0042] Hübschle, A. (2016). Security coordination in an illegal market: The transnational trade in rhinoceros horn. Politikon, 43(2), 193–214.

[cobi14262-bib-0043] Hübschle, A. M. (2017). The social economy of rhino poaching: Of economic freedom fighters, professional hunters and marginalized local people. Current Sociology, 65(3), 427–447.

[cobi14262-bib-0044] Jachmann, H. , & Billiouw, M. (1997). Elephant poaching and law enforcement in the Central Luangwa Valley, Zambia. Journal of Applied Ecology, 34(1), 233–244.

[cobi14262-bib-0045] Khan, S. N. (2014). Qualitative research method: Grounded theory. International Journal of Business and Management, 9(11), 224–233.

[cobi14262-bib-0046] Khanna, J. , & Harford, J. (1996). The ivory trade ban: Is it effective? Ecological Economics, 19(2), 147–155.

[cobi14262-bib-0047] Knapp, E. J. (2012). Why poaching pays: A summary of risks and benefits illegal hunters face in Western Serengeti, Tanzania. Tropical Conservation Science, 5(4), 434–445.

[cobi14262-bib-0048] Krishnasamy, K. , Milliken, T. , & Savini, C. (2016). In transition: Bangkok's ivory market—An 18‐month survey of Bangkok's ivory market. TRAFFIC. https://www.traffic.org/publications/reports/in‐transition‐bangkoks‐ivory‐market/

[cobi14262-bib-0049] Kuiper, T. , Altwegg, R. , Beale, C. , Carroll, T. , Dublin, H. T. , Hauenstein, S. , Kshatriya, M. , Schwarz, C. , Thouless, C. R. , Royle, A. , & Milner‐Gulland, E. J. (2023). Drivers and facilitators of the illegal killing of elephants across 64 African sites. Proceedings of the Royal Society B: Biological Sciences, 290(1990), Article 20222270. 10.1098/rspb.2022.2270 PMC9832558 36629103

[cobi14262-bib-0050] Leader‐Williams, N. (1996). Monitoring law enforcement and illegal activities. In K. Kangwana (Ed.), Studying elephants (pp. 148–161). African Wildlife Foundation.

[cobi14262-bib-0051] Leader‐Williams, N. , Albon, S. D. , & Berry, P. S. M. (1990). Illegal exploitation of black rhinoceros and elephant populations: Patterns of decline, law enforcement and patrol effort in Luangwa Valley, Zambia. Journal of Applied Ecology, 27(3), 1055–1087. 10.2307/2404395

[cobi14262-bib-0052] Leader‐Williams , N. , & Milner‐Gulland, E. J. (1993). Policies for the enforcement of wildlife laws: The balance between detection and penalties in Luangwa Valley, Zambia. Conservation Biology, 7(3), 611–617.

[cobi14262-bib-0053] Lemieux, A. M. , & Clarke, R. v. (2009). The international ban on ivory sales and its effects on elephant poaching in Africa. British Journal of Criminology, 49(4), 451–471.

[cobi14262-bib-0054] Lewis, D. , Kaweche, G. B. , & Mwenya, A. (1990). Wildlife conservation outside protected areas—Lessons from an experiment in Zambia. Conservation Biology, 4(2), 171–180.

[cobi14262-bib-0055] Lichtenstein, G. (2010). Vicuña conservation and poverty alleviation? Andean communities and international fibre markets. International Journal of the Commons, 4, 100–121.

[cobi14262-bib-0057] Lindsey, P. , Allan, J. , Brehony, P. , Dickman, A. , Robson, A. , Begg, C. , Bhammar, H. , Blanken, L. , Breuer, T. , Fitzgerald, K. , Flyman, M. , Gandiwa, P. , Giva, N. , Kaelo, D. , Nampindo, S. , Nyambe, N. , Steiner, K. , Parker, A. , Roe, D. , … Tyrrell, P. (2020). Conserving Africa's wildlife and wildlands through the COVID‐19 crisis and beyond. Nature Ecology & Evolution, 4(10), 1300–1310. 32728187 10.1038/s41559-020-1275-6

[cobi14262-bib-0058] Lindsey, P. A. , Miller, J. R. B. , Petracca, L. S. , Coad, L. , Dickman, A. J. , Fitzgerald, K. H. , Flyman, M. V. , Funston, P. J. , Henschel, P. , Kasiki, S. , Knights, K. , Loveridge, A. J. , MacDonald, D. W. , Mandisodza‐Chikerema, R. L. , Nazerali, S. , Plumptre, A. J. , Stevens, R. , van Zyl, H. W. , & Hunter, L. T. B. (2018). More than $1 billion needed annually to secure Africa's protected areas with lions. Proceedings of the National Academy of Sciences of the United States of America, 115(45), E10788–E10796. 30348785 10.1073/pnas.1805048115 PMC6233108

[cobi14262-bib-0059] Lindsey, P. A. , Romañach, S. S. , Tambling, C. J. , Chartier, K. , & Groom, R. (2011). Ecological and financial impacts of illegal bushmeat trade in Zimbabwe. Oryx, 45(1), 96–111.

[cobi14262-bib-0060] Lopes, A. A. (2015). Organized crimes against nature: Elephants in Southern Africa. Natural Resource Modeling, 28(1), 86–107.

[cobi14262-bib-0061] Martin, E. B. (1983). Rhino exploitation. World Wildlife Fund.

[cobi14262-bib-0062] Martin, R. B. (1993). Rhino population dynamics, illegal hunting and law enforcement in the lower Zambezi Valley in Zimbabwe. In O. A. Ryder (Ed.), Rhinoceros biology and conservation: Proceedings of an international conference (pp. 10–32). Zoological Society.

[cobi14262-bib-0063] Massé, F. , & Lunstrum, E. (2016). Accumulation by securitization: Commercial poaching, neoliberal conservation, and the creation of new wildlife frontiers. Geoforum, 69, 227–237.

[cobi14262-bib-0064] Maxwell, S. L. , Fuller, R. A. , Brooks, T. M. , & Watson, J. E. M. (2016). Biodiversity: The ravages of guns, nets and bulldozers. Nature, 536, 143–145. 27510207 10.1038/536143a

[cobi14262-bib-0309] Mbaiwa, J. E. (2018). Effects of the safarihunting tourism ban on rural livelihoods and wildlife conservation in Northern Botswana. South African Geographical Journal, 100(1), 41–61.

[cobi14262-bib-0065] McCarthy, D. P. , Donald, P. F. , Scharlemann, J. P. W. , Buchanan, G. M. , Balmford, A. , Green, J. M. H. , Bennun, L. A. , Burgess, N. D. , Fishpool, L. D. C. , Garnett, S. T. , Leonard, D. L. , Maloney, R. F. , Morling, P. , Schaefer, H. M. , Symes, A. , Wiedenfeld, D. A. , & Butchart, S. H. M. (2012). Financial costs of meeting global biodiversity conservation targets: Current spending and unmet needs. Science, 338(6109), 946–949. 23065904 10.1126/science.1229803

[cobi14262-bib-0066] Meijer, W. , Scheer, S. , Whan, E. , Yang, C. , & Kritski, E. (2018). Demand under the ban—China ivory consumption research post‐ban 2018. TRAFFIC & WWF.

[cobi14262-bib-0067] Messer, K. D. (2010). Protecting endangered species: When are shoot‐on‐sight policies the only viable option to stop poaching? Ecological Economics, 69(12), 2334–2340.

[cobi14262-bib-0068] Milledge, S. A. H. (2007). Illegal killing of African rhinos and horn trade, 2000–2005: The era of resurgent markets and emerging organized crime Illegal killing of African rhinos and horn trade, 2000–2005: The era of resurgent markets and emerging organized crime. Pachyderm, 43, 96–107.

[cobi14262-bib-0069] Milliken, T. (2014). Illegal trade in ivory and rhino horn: An assessment to improve law enforcement under the Wildlife TRAPS project. USAID and TRAFFIC.

[cobi14262-bib-0070] Milner‐Gulland, E. J. , & Leader‐Williams, N. (1992). A Model of incentives for the illegal exploitation of black rhinos and elephants: Poaching pays in Luangwa Valley, Zambia. Journal of Applied Ecology, 29(2), 388–401.

[cobi14262-bib-0071] Moher, D. , Liberati, A. , Tetzlaff, J. , & Altman, D. G. (2009). Preferred reporting items for systematic reviews and meta‐analyses: The PRISMA statement. BMJ, 339, Article b2535. 21603045 PMC3090117

[cobi14262-bib-0072] Mondol, S. , Mailand, C. R. , & Wasser, S. K. (2014). Male biased sex ratio of poached elephants is negatively related to poaching intensity over time. Conservation Genetics, 15, 1259–1263.

[cobi14262-bib-0073] Moyle, B. (2013). Conservation that's more than skin‐deep: Alligator farming. Biodiversity and Conservation, 22, 1663–1677.

[cobi14262-bib-0074] Mulero‐Pázmány, M. , Stolper, R. , van Essen, L. D. , Negro, J. J. , & Sassen, T. (2014). Remotely piloted aircraft systems as a rhinoceros anti‐poaching tool in Africa. PLoS ONE, 9(1), Article e83873. 24416177 10.1371/journal.pone.0083873 PMC3885534

[cobi14262-bib-0075] Mweetwa, T. , Christianson, D. , Becker, M. , Creel, S. , Rosenblatt, E. , Merkle, J. , Dröge, E. , Mwape, H. , Masonde, J. , & Simpamba, T. (2018). Quantifying lion (*Panthera leo*) demographic response following a three‐year moratorium on trophy hunting. PLoS ONE, 13(5), Article e0197030. 29782514 10.1371/journal.pone.0197030 PMC5962075

[cobi14262-bib-0076] Naidoo, R. , Weaver, L. C. , Diggle, R. W. , Matongo, G. , Stuart‐Hill, G. , & Thouless, C. (2016). Complementary benefits of tourism and hunting to communal conservancies in Namibia. Conservation Biology, 30(3), 628–638. 26537845 10.1111/cobi.12643

[cobi14262-bib-0077] Nanima, R. D. (2019). The Prevention of Organised Crime Act 1998: The need for extraterritorial jurisdiction to prosecute the higher echelons of those involved in rhino poaching. Potchefstroom Electronic Law Journal, 22(1), 1–46.

[cobi14262-bib-0078] Newing, H. , Watson, C. W. , Puri, R. K. , & Eagle, C. M. (2011). Conducting research in conservation: Social science methods and practice. Routledge.

[cobi14262-bib-0079] Nixon, S. , Pietersen, D. , Challender, D. , Hoffmann, M. , Godwill Ichu, I. , Bruce, T. , Ingram, D. J. , Matthews, N. , & Shirley, M. H. (2019). Smutsia gigantea . The IUCN Red List of Threatened Species, e.T12762A123584478. 10.2305/IUCN.UK.2019-3.RLTS.T12762A123584478.en

[cobi14262-bib-0080] Nowell, K. (2012). Wildlife crime scorecard: Assessing compliance with and enforcement of CITES commitments for tigers, rhinos and elephants . https://www.worldwildlife.org/publications/wildlife‐crime‐scorecard

[cobi14262-bib-0081] Olmedo, A. , Milner‐Gulland, E. J. , Challender, D. W. S. , Cugnière, L. , Dao, H. T. T. , Nguyen, L. B. , Nuno, A. , Potier, E. , Ribadeneira, M. , Thomas‐Walters, L. , Wan, A. K. Y. , Wang, Y. , & Veríssimo, D. (2020). A scoping review of celebrity endorsement in environmental campaigns and evidence for its effectiveness. Conservation Science and Practice, 2(10), Article e261.

[cobi14262-bib-0082] Olmedo, A. , Sharif, V. , & Milner‐Gulland, E. J. (2018). Evaluating the design of behavior change interventions: A case study of rhino horn in Vietnam. Conservation Letters, 11(1), Article e12365.

[cobi14262-bib-0085] Packer, C. , Loveridge, A. , Canney, S. , Caro, T. , Garnett, S. T. , Pfeifer, M. , Zander, K. K. , Swanson, A. , MacNulty, D. , Balme, G. , Bauer, H. , Begg, C. M. , Begg, K. S. , Bhalla, S. , Bissett, C. , Bodasing, T. , Brink, H. , Burger, A. , Burton, A. C. , … Polasky, S. (2013). Conserving large carnivores: Dollars and fence. Ecology Letters, 16(5), 635–641. 23461543 10.1111/ele.12091

[cobi14262-bib-0086] Partelow, S. , Schlüter, A. , von Wehrden, H. , Jänig, M. , & Senff, P. (2017). A sustainability agenda for tropical marine science. Conservation Letters, 11(1), Article e12351.

[cobi14262-bib-0087] Pietersen, D. , Jansen, R. , & Connelly, E. (2019). Smutsia temminckii . The IUCN Red List of Threatened Species 2019. 10.2305/IUCN.UK.2019

[cobi14262-bib-0089] Pimm, S. L. , Alibhai, S. , Bergl, R. , Dehgan, A. , Giri, C. , Jewell, Z. , Joppa, L. , Kays, R. , & Loarie, S. (2015). Emerging technologies to conserve biodiversity. Trends in Ecology and Evolution, 30(11), 685–696. 26437636 10.1016/j.tree.2015.08.008

[cobi14262-bib-0090] Rivalan, P. , Delmas, V. , Angulo, E. , Bull, L. S. , Hall, R. J. , Courchamp, F. , Rosser, A. M. , & Leader‐Williams, N. (2007). Can bans stimulate wildlife trade? Nature, 447(7144), 529–530. 17538599 10.1038/447529a

[cobi14262-bib-0091] Roe, D. , Dickman, A. , Kock, R. , Milner‐Gulland, E. J. , Rihoy, E. , & ’t Sas‐Rolfes, M. (2020). Beyond banning wildlife trade: COVID‐19, conservation and development. World Development, 136, Article 105121. 32834392 10.1016/j.worlddev.2020.105121 PMC7388857

[cobi14262-bib-0092] Rosenblatt, E. , Becker, M. S. , Creel, S. , Droge, E. , Mweetwa, T. , Schuette, P. A. , Watson, F. , Merkle, J. , & Mwape, H. (2014). Detecting declines of apex carnivores and evaluating their causes: An example with Zambian lions. Biological Conservation, 180, 176–186.

[cobi14262-bib-0093] Schlager, E. , & Ostrom, E. (1992). Property‐rights regimes and natural resources: A conceptual analysis. Land Economics, 68(3), 249–262.

[cobi14262-bib-0094] Schlossberg, S. , Chase, M. J. , Gobush, K. S. , Wasser, S. K. , & Lindsay, K. (2020). State‐space models reveal a continuing elephant poaching problem in most of Africa. Scientific Reports, 10(1), Article 10166. 32576862 10.1038/s41598-020-66906-w PMC7311459

[cobi14262-bib-0095] Schlossberg, S. , Chase, M. J. , & Sutcliffe, R. (2019). Evidence of a growing elephant poaching problem in Botswana. Current Biology, 29(13), 2222–2228. 31204160 10.1016/j.cub.2019.05.061

[cobi14262-bib-0096] Selier, S. A. J. , Page, B. R. , Vanak, A. T. , & Slotow, R. (2013). Sustainability of elephant hunting across international borders in southern Africa: A case study of the greater Mapungubwe Transfrontier Conservation Area. The Journal of Wildlife Management, 78(1), 122–132.

[cobi14262-bib-0097] Shepherd, C. R. , Connelly, E. , Hywood, L. , & Cassey, P. (2017). Taking a stand against illegal wildlife trade: The Zimbabwean approach to pangolin conservation. Oryx, 51(2), 280–285.

[cobi14262-bib-0098] Siamudaala, V. M. , Nyirenda, V. R. , & Saiwana, L. M. (2009). Effectiveness of law enforcement on wildlife crimes in the Kafue ecosystem in Zambia. ZAWA.

[cobi14262-bib-0099] Sibanda, M. , Dube, T. , Bangamwabo, V. M. , Mutanga, O. , Shoko, C. , & Gumindoga, W. (2016). Understanding the spatial distribution of elephant (*Loxodonta africana*) poaching incidences in the mid‐Zambezi Valley, Zimbabwe using geographic information systems and remote sensing. Geocarto International, 31(9), 1006–1018.

[cobi14262-bib-0100] Soliku, O. , & Schraml, U. (2018). Making sense of protected area conflicts and management approaches: A review of causes, contexts and conflict management strategies. Biological Conservation, 222, 136–145.

[cobi14262-bib-0101] Somerville, K. (2016). Ivory: Power and poaching in Africa (1st ed.). Hurst.

[cobi14262-bib-0102] Stiles, D. (2004). The ivory trade and elephant conservation. Environmental Conservation, 31(4), 309–321.

[cobi14262-bib-0201] Strong, M. , & Silva, J. A. (2020). Impacts of hunting prohibitions on multidimensional well‐being. Biological Conservation, 243, Article 108451.

[cobi14262-bib-0202] Swanson, T. M. (1994). The Economics of Extinction Revisited and Revised: A Generalised Framework for the Analysis of the Problems of Endangered Species and Biodiversity Losses. *Oxford Economic Papers*, *46*(Supplement_1), 800–821.

[cobi14262-bib-0203] Swanson, T. M. (1995). The Economics and Ecology of Biodiversity Decline: The Forces Driving Global Change. Cambridge University Press.

[cobi14262-bib-0204] Tanghe, P. F. (2017). When rhinos *are sacred: Why some countries control poaching* [University of Denver].

[cobi14262-bib-0205] Taylor, M. (2007). CBNRM for whose benefit? A case study of subsistence hunting on the boundaries of Botswana's northern Protected Areas. In: Schuster, B. & Thakadu, O. T. (Eds.), Natural Resources Management and People (pp. 27–33). IUCN CBNRMSupport Programme.

[cobi14262-bib-0206] Thorbjarnarson, J. (1999). Crocodile tears and skins: International trade, economic constraints, and limits to the sustainable use of crocodilians. Conservation Biology, 13, 465–470.

[cobi14262-bib-0207] Tilman, D. , Clark, M. , Williams, D. R. , Kimmel, K. , Polasky, S. , & Packer, C. (2017). Future threats to biodiversity and pathways to their prevention. Nature, 546(7656), 73–81. 28569796 10.1038/nature22900

[cobi14262-bib-0208] ′t Sas‐Rolfes, M. , & Hiller, C. (2020). Literature Review: Assessment of the Impact of Trade Restrictions and Other Policies on Wildlife Conservation and Community Wildlife Stewardship in Southern Africa. https://biodiversitylinks.org/projects/mission‐projects/vukanow/resources/trade‐restrictions‐low‐res‐spreadsfinal.pdf

[cobi14262-bib-0209] ′t Sas‐Rolfes, M. (2000). Assessing CITES: four case studies. In: Hutton, J. & Dickson, B. (Eds.), Endangered Species, Threatened Convention (pp. 69–87). Earthscan Publications.

[cobi14262-bib-0210] ′t Sas‐Rolfes, M. (2012). TheRhino Poaching Crisis: A Market Analysis . http://www.rhinoresourcecenter.com/pdf_files/133/1331370813.pdf

[cobi14262-bib-0211] ′t Sas‐Rolfes, M. (2017). African wildlife conservation and the evolution of hunting institutions. Environmental Research Letters, 12(11), Article 115007.

[cobi14262-bib-0212] ′t Sas‐Rolfes, M. , Challender, D. W. , Hinsley, A. , Veríssimo, D. , & Milner‐Gulland, E. (2019). Annual Review of Environment and Resources Illegal Wildlife Trade: Scale, Processes, and Governance. Annual Review of Environment and Resources, 44, 201–228.

[cobi14262-bib-0213] ′t Sas‐Rolfes, M. , Emslie, R. , Adcock, K. , & Knight, M. (2022). Legal hunting for conservation of highly threatened species: The case of African rhinos. Conservation Letters, 15(3), Article e12877.

[cobi14262-bib-0214] ′t Sas‐Rolfes, M. , Moyle, B. , & Stiles, D. (2014). The complex policy issue of elephant ivory stockpile management. Pachyderm, 55, 62–77.

[cobi14262-bib-0215] Underwood, F. M. , Burn, R. W. , & Milliken, T. (2013). Dissecting the illegal ivory trade: An analysis of ivory seizures data. PLoS ONE, 8(10), Article e76539. 24250744 10.1371/journal.pone.0076539 PMC3799824

[cobi14262-bib-0216] UNEP . (2019). Policy Brief. Effectiveness of policy interventions relating to the illegal and unsustainable wildlife trade. United Nations Environment Programme . www.unenvironment.org

[cobi14262-bib-0217] Veríssimo, D. , & Wan, A. K. Y. (2019). Characterizing efforts to reduce consumer demand for wildlife products. Conservation Biology, 33(3), 623–633. 30259569 10.1111/cobi.13227

[cobi14262-bib-0218] Vundla, N. L. (2019). *Mangalanecommunity's perceptions of poverty as factors influencing involvement in rhino poaching: A case study of Mozambique* [Stellenbosch University].

[cobi14262-bib-0219] Wasser, S. K. , & Gobush, K. S. (2019). Conservation: Monitoring elephant poaching to prevent a population crash. Current Biology, 29(13), R627–R630. 31386839 10.1016/j.cub.2019.07.029

[cobi14262-bib-0220] White, P. A. , & Belant, J. L. (2015). Provisioning of game meat to rural communities as a benefit of sport hunting in Zambia. PLoS ONE, 10(2), Article e0117237. 25693191 10.1371/journal.pone.0117237 PMC4334497

[cobi14262-bib-0221] Wijnstekers, W. (2018). The Evolution of CITES: A reference to the Convention on International Trade in Endangered Species of Wild Fauna and Flora (11th ed.). CIC ‐ InternationalCouncil for Game and Wildlife Conservation.

[cobi14262-bib-0222] Williams, V. L. , Coals, P. G. , de Bruyn, M. , Naude, V. N. , Dalton, D. L. , & Kotzé, A. (2021). Monitoring compliance of CITES lion bone exports from South Africa. PLOS ONE, 16(4), Article e0249306. 33798210 10.1371/journal.pone.0249306 PMC8018656

[cobi14262-bib-0223] Williams, V. L. , Loveridge, A. J. , Newton, D. J. , & Macdonald, D. W. (2017a). A roaring trade? The legal trade in Panthera leo bones from Africa to East‐Southeast Asia. PLoS ONE, 12(10), Article e0185996. 29065143 10.1371/journal.pone.0185996 PMC5655489

[cobi14262-bib-0224] Williams, V. L. , Loveridge, A. J. , Newton, D. J. , & Macdonald, D. W. (2017b). Questionnaire survey of the pan‐African trade in lion body parts. PLoS ONE, 12(10), Article e0187060. 29073202 10.1371/journal.pone.0187060 PMC5658145

[cobi14262-bib-0225] Williams, V. L. , & ’tSas‐Rolfes, M. J. (2019). Born captive: A survey of the lion breeding, keeping and hunting industries in South Africa. PLOS ONE, 14(5), Article e0217409. 31136596 10.1371/journal.pone.0217409 PMC6538166

[cobi14262-bib-0226] Wilson, E. O. (1989). Threats to biodiversity. Scientific American, 261(3), 108–117.

[cobi14262-bib-0227] Witter, R. , & Satterfield, T. (2019). Rhino poaching and the “slow violence” of conservation‐related resettlement in Mozambique's Limpopo National Park. Geoforum, 101, 275–284.

[cobi14262-bib-0228] Zhou, X. , Wang, Q. , Zhang, W. , Jin, Y. , Wang, Z. , Chai, Z. , Zhou, Z. , Cui, X. , & MacMillan, D. C. (2018). Elephant poaching and the ivory trade: The impact of demand reduction and enforcement efforts by China from 2005 –2017. Global Ecology and Conservation, 16, Article e00486.

