# Supplementary material for: Systematic review of the impact of restrictive wildlife trade measures on conservation of iconic species in southern Africa
Source: Conserv Biol. 2024 Apr 5;39(1):e14262. doi: 10.1111/cobi.14262 (PMC11780195; doi:10.1111/cobi.14262)
Supplement: Supplementary file 1 — Appendix S1: List of databases and organizations for grey literature search, including successful search terms per website Appendix S2: Review and data extraction protocol applied to eligible studies after applying the selection criteria [file COBI-39-e14262-s001.pdf]

Appendix S1: *List of databases and organizations for grey literature search, including successful search terms per website*

Organisation	Link	Successful Search Terms
**International Impact Initiative (3ie)**	www.3ieimpact.org	“wildlife crime”, “wildlife product*”, “illegal wildlife trade”, “illicit wildlife trade”, “traffic*”, “poach*”
**Collaboration for Environmental Evidence**	www.environmentalevidence.com	“wildlife”
**International Institute for Environment and Development (IIED)**	www.iied.org	“Wildlife Crime”, “Illegal Wildlife Trade”, “Poaching”
**Wildlife Conservation Society (WCS)**	https://global.wcs.org/Resources/Publications.aspx	“TRADE”
**TRAFFIC, the wildlife trade monitoring network**	www.traffic.org	“Wildlife crime”, “Illegal wildlife”, “Illegal trade”, “Illicit wildlife”, “Illicit trade”, “Poaching”
**Worldwide Fund for Nature Conservation (WWF)**	https://wwf.panda.org/knowledge_hub/all_publications/	“wildlife crime”, “Illegal wildlife”, “Illegal trade”, “illicit trade”, “poaching”
**United Nations Environment Programme – World Conservation Monitoring Centre (UNEP-WCMC)**	www.unep-wcmc.org	“Trade”
**International Union for the Conservation of Nature (IUCN)**	www.iucn.org	“Crime”, “illegal trade”, “Poaching”
**The Convention on International Trade in Endagered Species of Wildlife Fauna and Flora (CITES)**	www.cites.org<br>Searched via Google Scholar; search term site:cites.org	“wildlife crime” AND Africa site:cites.org, “illegal wildlife” AND Africa site:cites.org, “illicit wildlife” site:cites.org, “illegal trade” site:cites.org, “illicit trade” site:cites.org, “poaching” site:cites.org
**The Convention on Biological Diversity (CBD)**	www.cbd.int<br>Searched via Google Scholar; search term site:cites.org	"Wildlife Crime" AND Africa site:cbd.int; “illegal wildlife” site:cbd.int, “illicit wildlife” site:cbd.int, “illicit trade” site:cbd.int, “illegal trade” site:cbd.int, “poaching” site:cbd.int
**Conservation Evidence**	www.conservationevidence.com	“Poaching”, “Trade”, “Illegal”
**United States Agency for International Development (USAID)**	www.usaid.org	"Wildlife trade“, "Poaching“, “Wildlife trade"
**Rhino Resource Center**	http://www.rhinoresourcecenter.com/index.php?s=1&act=refs	“Illegal wildlife”, “Illicit wildlife”, “Wildlife crime”, “Illicit trade”, “Illegal trade”, “Poaching”

Appendix S2: *Review and data extraction protocol applied to eligible studies after applying the selection criteria*

IMPACT ASSESSMENT TRADE RESTRICTIONS on WILDLIFE CONSERVATION and COMMUNITY WILDLIFE STEWARDSHIP
CODING SHEET

				Data Type	Categories / Notes
0	General				
	0.1	UID	Publication Unique Identifier (UID)	Text	Combi: 3 Letters Main Author, 3 Lett Titel, 3 numbers 001…
	0.2	AssName	Name of Assessor	Text	Initials (TH, MSR)
	0.3	AssDate	Date of Publication Assessment	Date	TTMMYYYY
1	Bibliographic Information				
	1.1	LitTyp	Literature Type (Peer-Review vs Grey)	List	Peer-Reviewed; Grey
	1.2	PubTyp	Publication Type	List	Book; Chapter; Article; Report; Review; Dissertation; Other
	1.3	AU	Author(s)	Text	
	1.4	TI	Title	Text	
	1.5	SRC	Source/Journal	Text	
	1.6	PubYear	Year of Publication	Date	YYYY
	1.7	Link	Access Link	Text	doi-Link or Website-Link for Download
	1.8	DB	Database/Website Search	List	Web of Science; Scopus; Google Scholar; Grey Literature Websites, Referenced
2	Assessment of Relevance				
	2.1	Decision	Decision	List	Y/N
	2.2	Rejection	Reason for Rejection	List	1) irrelevant study topic<br>2) irrelevant influencing factor<br>3) irrelevant outcome variable<br>4) irrelevant location<br>5) Lack of evidence (counterfactuals)<br>6) inept study design (modelling, editorial, commentary, …)<br>7) Other (e.g. duplicate, language, missing document,…)
3	Basic Information on Study				
	3.1	PolType	Type of trade restriction or other policy	Text	1) Legal supply restrictions (area- or species-based); e.g. trade bans, quotas, moratoriums, etc.<br>4) Monitoring & enforcing of restrictions<br>5) Legal Trade Regulations (e.g. CITES)<br>6) Campaign directly linked to consumption regulation<br>7) Identifying and disrupting actor-networks and their enabling context (e.g. social network analysis and financial transaction analysis)
	3.2	Goal	Stated goals of study / main research question	Text	
	3.3	TVar	Input Variable(s)	Text	
	3.4	Chain	Where along the supply chain does this study aim?	List	Supply-Side; Transactional; Demand-Side
	3.5	Country	Country/Region	List	Angola; Botswana; Namibia; Malawi; Mozambique; South Africa; Tanzania; Zambia; Zimbabwe; SeveralSADC; AllSADC
	3.6	Scale	Spatial Scale of Study	List	local, regional, national, trans-national, SADC-wide
	3.7	Species	Species	Text	
	3.8	ResAff	Affiliation of Research (NGO, University, Other)	List	NGO, University, Government/Regulatory Agency, Other
	3.9	StudDes	Type of study / study design	List	e.g.: before/after, presence/absence, control/impact, multi-regression modelling, review, etc.
	3.10	DataType	Type of data	List	Qualitative, Quantitative, Mixed
	3.11	Meth	Study method	Text	
	3.12	CounFac	Robustness check performed	List	counterfactual thinking: Y/N
	3.13	StudCon	Does the study inlcude a control?	List	Y/N
	3.14	Tscal	Duration of study / Temporal Scale	Number	in months
4	Information on Outcomes				
	4.1	Find	Stated main outcomes / findings	Text	Summary of main finding(s)
	4.2	ResType	Outcome variable categories	List + Notes	Behaviour (Wildlife Crime Statistics like seizures, poaching numbers, conviciton rates; attitude towards wildlife crime, price data where available, demand reduction); Biology (Species/Population Conservation); Mixed; Other
	4.3	OVar	Outcome variable(s)	Text	
	4.4	OData	Data Type of Outcome	List	Qualitative, Quantitative, Mixed
	4.5	ImpType	Type of Impact (Positive, negative, neutral, mixed)	List	Positive; Negative; Neutral; Mixed
	4.6	Confound	Confounds / Special Circumstances	Text	Comment on special cirumstances when appropriate
	4.7	Recomm	Learning / Recommendation	Text	Comment on recommendations when approporiate

Appendix S3: *Data analysis results showing emergent clusters for specific levers linked to legal measures to restrict supply with evidenced effects on direct and indirect wildlife conservation outcomes as well as rural livelihood/well-being and national economies*

Impact assessments of legal restrictions to reduce supply (* italic = peripheral; * grey = duplicate)

**Lever/Determinant**	**Impact**	**Effect**	**Impact Assessment Studies**	**Species**	**Country**
Wildlife hunting ban or moratorium	negative	Control of illegal hunting/poaching	(Gaodirelwe et al., 2020a)<br>(Gaodirelwe et al., 2020b)<br>(Schlossberg et al., 2019)	General wildlife<br>General wildlife<br>Elephant	Botswana<br>Botswana<br>Botswana
	positive	Stabilisation/growth of threatened species populations	(Mweetwa et al., 2018)<br>(Rosenblatt et al., 2014)	Lion<br>Lion	Zambia<br>Zambia
	negative	Rural livelihood and well-being	(Gaodirelwe et al., 2020a)<br>(Strong and Silva, 2020)<br>(White and Belant, 2015)<br>(*Mbaiwa, 2018*)<br>(*Taylor, 2007*)	General wildlife<br>General wildlife + Rhino<br>General wildlife<br>General wildlife<br>General wildlife	Botswana<br>Mozambique & Namibia<br>Zambia<br>Botswana<br>Botswana
Bonus-malus hunting system	positive	Stabilisation/growth of threatened species populations	(Begg et al., 2018)	Lion	Mozambique
Uncoordinated hunting quotas across national borders	negative	Stabilisation/growth of threatened species populations	(Selier et al., 2013)	Elephant	Botswana, South Africa, Zimbabwe

Appendix S4: *Data analysis results showing emergent clusters for specific levers linked to law enforcement measures with evidenced effects on direct and indirect wildlife conservation outcomes as well as rural livelihood/well-being and national economies*

Impact assessments of law enforcement measures (* blue = modelling approaches; * italic = peripheral; * grey = duplicate)

Lever/Determinant	Impact	Effectiveness	Impact Assessment Studies	Species	Country
Sufficient, capable patrol resources (chance of detection)	positive	Control of illegal hunting/poaching	(Ball et al., 2018)<br>(Lopes, 2015)<br>(Di Minin et al., 2015)<br>(Gandiwa et al., 2013)<br>(Burn et al., 2011)<br>(Siamudaala et al., 2009)<br>(Leader-Williams, 1996)<br>(Leader-Williams et al., 1990)<br>(Lewis et al., 1990)<br>*(Milledge, 2007)*	Rhino<br>Elephant<br>Rhino<br>General wildlife<br>Elephant<br>Elephant, Buffalo , Puku<br>Elephant & Rhino<br>Elephant, Rhino<br>General wildlife<br>Rhino	Zimbabwe<br>Southern Africa<br>South Africa<br>Zimbabwe<br>Africa-wide<br>Zambia<br>Zambia<br>Zambia<br>Zambia<br>Africa-wide
	positive	Stabilisation/growth of threatened species populations	(Packer et al., 2013)<br>(Jachmann and Billiouw, 1997)<br>(Leader-Williams et al., 1990)<br>(Leader-Williams, 1996)	Lion<br>Elephant<br>Elephant & Rhino<br>Elephant & Rhino	Zambia<br>Zambia<br>Zambia<br>Zambia
	ambiguous	Control of illegal hunting/poaching	(Hauenstein et al., 2019)<br>(Barichievi et al., 2017)<br>(Knapp, 2012)<br>(*CITES Secretariat, 2007*)<br>(Martin, 1993)	Elephant<br>Rhino<br>General wildlife<br>Rhino	Africa-wide<br>South Africa<br>Tanzania<br>Zimbabwe
	negative	Rural livelihood and well-being	(*Vundla, 2019*)<br>(Witter and Satterfield, 2019)<br>(Hübschle, 2017)<br>(*Massé and Lunstrum, 2016*)	Rhino + high-value species<br>Rhino<br>Rhino<br>Rhino	Mozambique<br>Mozambique<br>South Africa<br>Mozambique
Fines / Sentences varying with poaching output	positive	Control of illegal hunting/poaching	(Shepherd et al., 2017)<br>(Di Minin et al., 2015)<br>(Leader-Williams and Milner-Gulland, 1993)<br>(Milner-Gulland and Leader-Williams, 1992)<br>(Messer, 2010)	Pangolin<br>Rhino<br>Elephant, Rhino<br>Rhino & Elephant<br>General wildlife	Zimbabwe<br>South Africa<br>Zambia<br>Zambia<br>Africa-wide
Animal population monitoring	positive	Control of illegal hunting/poaching & stabilisation of threatened species populations	(Ferreira et al., 2017)<br>(Bauer et al., 2015)	Rhino<br>Lion	South Africa<br>Africa-wide
Detection-improving technology	positive	Detection probability of poachers	(*Bondi et al. 2018*), (*Sibanda et al., 2016*), (*Hart et al., 2015*), (*Mondol et al., 2014*), (*Mulero-Pázmány et al., 2014*)		
Non-market related social construct (morale outrage)	positive	Control of illegal hunting/poaching	(Tanghe, 2017)	Rhino	South Africa, Swaziland, Nepal

Appendix S5: *Data analysis results showing emergent clusters for specific levers linked to international trade regulations to reduce supply with evidenced effects on direct and indirect wildlife conservation outcomes as well as rural livelihood/well-being and national economies*

Impact assessments of international trade regulations to reduce supply (* blue = modelling approaches; * italic = peripheral)

Lever/Determinant	Impact	Effectiveness	Impact Assessment Studies	Species	Country
International trade ban; Appendix I listing	negative	Stabilisation/growth of threatened species populations	(*Ferreira et al., 2018*)<br>(*Emslie et al., 2016*)<br>(de Beer, 2016)<br>(*Cheteni, 2014*)<br>(*Underwood et al., 2013*)	Rhino<br>Rhino<br>Rhino<br>Rhino<br>Elephant	South Africa<br>Africa-wide<br>South Africa<br>Africa-wide<br>Africa-wide
Announcement of international trade bans; zero quotas	negative	Stabilisation of trade numbers to sustainable levels	(*Williams and 't Sas-Rolfes, 2019*)<br>(Williams et al., 2017a)<br>(*Williams et al., 2017b*)<br>(*Challender et al., 2015*)	Lion<br>Lion<br>Lion<br>Pangolin	South Africa<br>South Africa<br>South Africa<br>*Asia*
International trade ban / Appendix I Listing with post-ban regulated ivory market	positive	Stabilisation/growth of threatened species populations	(Zhou et al., 2018)<br>(Lemieux and Clarke, 2009)<br>(Stiles, 2004)<br>(Heltberg, 2001)<br>(*Bulte and van Kooten, 1999*)<br>(Khanna and Harford, 1996)	Elephant<br>Elephant<br>Elephant<br>Elephant<br>Elephant<br>Elephant	Africa-wide<br>Africa-wide<br>Africa-wide<br>Africa-wide<br>Zambia<br>Africa-wide
International trade ban; Appendix I listing	negative	National economies	(Barnes, 1996)	Elephant	Botswana
One-off sales	mixed	Control of illegal hunting/poaching	(Bulte et al., 2007)	Elephant	Africa-wide

Appendix S6: *Data analysis results showing emergent clusters for specific levers linked to measures to identify and disrupt actor networks with evidenced effects on direct and indirect wildlife conservation outcomes as well as rural livelihood/well-being and national economies*

Identification and Disruption of Actors-networks (* italic = peripheral)

Lever/Determinant	Impact	Effectiveness	Impact Assessment Studies	Species	Country
Social network analysis	postitive	Actionable intelligence reports to disrupt criminal networks	(Haas and Ferreira, 2015)	Rhino	South Africa
Missing augmentation of intelligence with risk assessment activities	negative	Successful investigations, prosectution and conviction	(*Milliken, 2014*)<br>(*Hübschle, 2016*)	Elephant, Rhino<br>Rhino	Africa-wide<br>South Africa
Lack of extraterritorial jurisdiction	negative	Successful investigations, prosectution and conviction	(*Nanima, 2019*)<br>(Hübschle, 2017)	Rhino<br>Rhino	South Africa<br>South Africa

Appendix S7: *Data analysis results showing emergent clusters for specific levers linked to legal measures to reduce demand with evidenced effects on demand in the form of a simultaneous decline in both consumer sales volumes and prices*

Impact assessments of legal restrictions to reduce demand (* blue = modelling approaches; * italic = peripheral; * grey = duplicate)

Lever/Determinant	Impact	Effectiveness	Impact Assessment Studies	Species	Country
Ban of import, export, trade and sale	indirect, positive	Reduction of legal trade	(Krishnasamy et al., 2016)	Elephant	Thailand
	indirect, positive	Increase in purchase rejectors; negative attitude towards purchase (stigmatisation)	(Meijer et al., 2018)<br>(*Challender et al., 2015*)	Elephant	China
Government restrictions and corresponding campaign programs	indirect, positive	Fall in prices for consumers	(Zhou et al., 2018)	Elephant	China
Government restrictions and corresponding campaign programs	indirect, positive	Stigmatisation of purchases	(Zhou et al., 2018)<br>(Stiles, 2004)	Elephant<br>Elephant	China<br>Africa-wide
Improved legal governance in consumer countries	positive	Control of illegal hunting/poaching	(Di Minin et al., 2015)	Rhino	Viet Nam, Thailand
